# Structural uncertainty in mapping Euro-Atlantic atmospheric rivers obscures understanding of associated meteorological extremes

**DOI:** 10.1038/s41598-025-19685-1

**Published:** 2025-09-29

**Authors:** Venugopal Thandlam, Anna Rutgersson, Erik Sahlée

**Affiliations:** 1 Air, Water and Landscape Science (LUVAL), Department of Earth Sciences, Uppsala University, Uppsala, Sweden; 2 Centre of Natural Hazards and Disaster Science (CNDS), Uppsala University, Uppsala, Sweden; 3 The Center for Environment and Development Studies Research Forum (CEFO), Uppsala University, Uppsala, Sweden

**Keywords:** Atmospheric rivers, TECA-BARD, Structural uncertainty, IVT, AR probabilities, Deciles, Europe, Meteorological extremes, Climate sciences, Atmospheric science

## Abstract

Understanding uncertainties in meteorological extremes induced by Atmospheric river (AR) structural uncertainties can help to develop effective strategies to mitigate AR induced hazards and adapt to changing climate conditions. As a first step, this study examines the statistical relationship between AR structural uncertainty and the characterisation of associated meteorological extremes over the Euro-Atlantic region, using long-term historical data from ECMWF Reanalysis v5 (ERA5) during 1940 to 2022. Leveraging the Bayesian AR detection (BARD), a form of statistical machine learning model in the Toolkit for Extreme Climate Analysis (TECA), we examine the impact of structural uncertainties in AR dimensions on daily precipitation (wet), wind speeds (windy), and temperature (warm/cold) anomalies and extremes over Europe, the UK and Scandinavia. A large spread in the aggregated detected AR probabilities (ARP) spatially and temporally led to differences in ARs’ attributes, such as frequency, integrated water vapour transport (IVT; intensity), and their impact on weather parameters, anomalies and extremes at selected probability thresholds across space and time. The magnitude of AR impacts and associated meteorological phenomena over land varies based on the chosen deciles (dividing ARP into ten equal parts with a 0.1 increase) of ARPs, along with the default threshold from the model ($ARP \ge 0.67$). AR intensities and landfalling area are increasing over the study period, irrespective of the selected ARP. The effects of AR structural uncertainties are more prominent over inland Europe and Scandinavia than over coastal Europe and the UK. The physical and meteorological phenomena underlying these results require further exploration to understand the impact of landfalling ARs on land.

## Introduction

A range of weather phenomena such as tropical and extratropical cyclones, fronts, mesoscale convective systems, and atmospheric rivers (ARs) influence precipitation and other meteorological processes over land^1–6^. ARs are long, narrow, and intense bands of moisture that transport vast quantities of water vapour from the tropics to higher latitudes^7^. These atmospheric conduits influence regional precipitation patterns and extreme weather events^8^. ARs, in particular, have contributed a great deal to our understanding of the water cycle^9^, precipitation extremes^8,10,11^ and variability^12^, impacts^13–15^, atmospheric dynamics^16^, cryospheric changes^17–19^, and uncertainty in projections of precipitation in future climate change scenarios^10,11,20,21^.

Hence, accurate detection and tracking of ARs are essential for understanding their impacts on weather and climate and for developing effective mitigation and adaptation strategies^22^. However, ARs are notoriously tricky to detect and track due to their subtle and dynamic nature^7^. Numerous detection algorithms have been developed, each with strengths and weaknesses, leading to discrepancies in reported AR frequencies, duration, and intensities^7,23^. Additionally, the inherent variability of ARs, influenced by various factors such as atmospheric dynamics, land-sea configurations, and upper-level winds^24–26^, further complicates their quantification. These uncertainties in AR detection pose challenges to understanding their associated extreme weather events^7^.

Over the past decade, the number of methods used to detect ARs has increased. In the last 5 years, there has been a growing understanding that uncertainty in AR detection may impact our scientific knowledge. To assess this impact, the Atmospheric River Tracking Method Intercomparison Project (ARTMIP) was created^27^. Through a series of controlled, collaborative experiments, ARTMIP has shown that some aspects of our understanding of AR-related science depend on detector design and the detection algorithm used,^7,27^. ARTMIP has made significant efforts to quantify uncertainty, and the community has produced several important papers on this topic. However, it would be impractical to perform ARTMIP-like experiments for every AR-related scientific question that arises. This raises the question of how to best deal with uncertainty in AR detection practically^18,28^.

O’Brien et al.^29^ discuss and address the challenges of uncertainty quantification and optimisation by developing a formal Bayesian framework that samples ’plausible’ sets of parameters from an AR detector. A database of AR counts constrains the Bayesian method. The AR detectors designed from expert labels include only three main criteria: contiguity above a threshold, size, and location, whose ranges are described in Table 1^29^. Thus, the Toolkit for Extreme Climate Analysis Bayesian AR Detector (TECA-BARD) performs similarly to an ensemble of algorithms from ARTMIP, emulating the counting statistics of the contributors who provided AR counts. The analysis of the impact of the El Niño-Southern Oscillation (ENSO) on AR predictability on a regional scale yields conclusions that differ from those of Guan and Waliser^30,31^. Different AR detection algorithms used by Goldenson et al. ^31^ and Guan and Waliser^30^ might have caused this disparity in the inferred relationships between ENSO and ARs, which can be removed by using methods like TECA-BARD.

**Table 1:** Ranges and priors in the AR detectors used in TECA-BARD.

Description	Range
Percentile threshold for integrated water vapour transport (IVT; unitless)	80^th^ to 99^th^ (0.8, 0.99)
Size: Minimum area of contiguous region	(1 $\times$ 10^11^, 5 $\times$ 10^12^) m$^2$
Location: Zonal half width at half maximum of tropical filter	(5, 25)$^\circ$ N

The percentile threshold for integrated water vapour transport (IVT; unitless) indicates the rank of IVT values within a distribution, not the actual measurements. For example, the 80th percentile would mean that 80% of the IVT values in the dataset are lower than this value, while 20% are higher. Unlike other algorithm outputs in ARTMIP^27^, TECA-BARD does not provide a binary indicator of AR presence but rather a posterior probability of AR detection (ARP). However, the developers used $\frac{2}{3}$ probability (0.67) globally as a minimum and default threshold to indicate the AR binary tag (ARBT), denoted as AR. This is analogous to previous studies using heuristic approaches in threshold selection^7,30^. However, here, the heuristics lie in the aggregated probability from 1024 AR detectors, rather than the selections or settings made using the physical parameters of ARs.

To compare TECA-BARD’s output with other ARTMIP algorithms, developers have created a comparable measure of AR presence by averaging binary AR identifications across available algorithms in ARTMIP on a location-by-location basis globally. The comparison was made between January 1, 1980, and June 30, 2017, using MERRA-2 reanalysis data^32^. The comparison showed that ARTMIP and TECA-BARD agree on the presence of “high confidence” ARs. There is less agreement over the eastern United States and the central North Atlantic, where ARTMIP scores exceed 0.9, and TECA-BARD’s score is approximately 0.6. These regions are relatively small areas with high IVT. According to O’Brien et al.^29^, this behaviour occurs due to multimodality in the posterior distribution of parameters, which shows several distinct modes in the minimum area parameter.

Similarly, the study disagreed with ARTMIP and TECA-BARD on the accuracy of certain regions with a “low confidence index”. The main disagreement is in the tropics, where ARTMIP has a probability of approximately 0.2 (20%). In contrast, TECA-BARD has a probability of roughly zero throughout the tropics due to the filtering and masking applied. The disagreement is attributed to ARTMIP incorrectly detecting the Inter-Tropical Convergence Zone by a small number of algorithms. TECA-BARD has a filter to avoid such an incorrect detection in the tropics. Although the model addressed the reduction of uncertainties in threshold-based AR mapping, several factors have not yet been explored.

1. Using AR counts instead of AR footprints is potentially a limitation of the study by O’Brien et al.^29^.
2. However, developers applied the model globally to count ARs without focusing on the regional perspectives of their footprints. Thus, there was no attempt to use the TECA-BARD regionally to estimate the uncertainty in mapping ARs and AR-induced wet, windy, and warm/cold anomalies and extremes.
3. Also, it is essential to investigate how the less agreement between TECA-BARD (0.6) and ARTMIP (0.9) on the presence of “low-confidence” ARs over the narrow region of the North Atlantic (with high IVTs) would impact the AR-induced wet, windy, and warm/cold anomalies and extremes over Europe, the UK and Scandinavia.
4. Thus, the impact of detection uncertainty due to the multimodality of detection parameters on regional meteorological extremes over land has not been explored previously.
5. Similarly, the current study examines the validity of the default probability ($\ge$0.67), i.e., ARBT (AR regions with lower uncertainty), on understanding AR-associated wet, windy, and warm/cold anomalies and extremes over Europe, compared to ARP as a whole with higher uncertainty (regions including lower probabilities). All these aspects prompt us to use and check the framework over the Euro-Atlantic region to study the impact of uncertainty in mapping ARs on wet, windy, and warm/cold anomalies and extremes over Europe, the UK and Scandinavia.

We briefly explained the data used in Section 2. Results are shown in Section 3 on mapping ARs, a use case for uncertainty quantification using TECA-BARD in the pan-Atlantic region. As it is impossible to look after all the ARPs ranging from 0-1, we present results with ARP, representing all probabilities and larger uncertainty, and ARBT, representing AR with lesser uncertainty. Thus, the results include the spatiotemporal dependence of Euro-Atlantic ARs on ARP and ARBT, as well as associated changes in frequencies. However, the work also highlights the impact of detection uncertainty by selecting ARP deciles on the variability of AR characteristics and meteorological variables. These deciles divide the ARP into ten equal parts (with 0.1 intervals) to understand the reduced uncertainty on the AR area covered, associated IVT, and wet, windy, and warm/cold anomalies and extremes. We present simultaneous Pearson correlation coefficients (hereafter, ’correlation’) and spatiotemporal (annual) regression coefficients to study the relationship among ARs (with ARP and ARBT) and area, IVT and meteorological parameters, including their compoundness and concurrency of occurrence. Section 4 discusses possible physical and meteorological mechanisms that lead to the results. We summarise and conclude the results in Section 5, including the study’s limitations.

## Data and methods

We use ECMWF Reanalysis v5 (ERA5) 6-hourly instantaneous data^33^ of specific humidity, zonal, and meridional wind components available during 1940–2020 at 0.25-degree horizontal resolution at all available vertical levels from 1000 to 300 hPa. This data, obtained from the climate data store (CDS, https://cds.climate.copernicus.eu/), is used as input to TECA-BARD to map the ARs with inline uncertainty over the Euro-Atlantic region (100 W–25 E; 0–80 N). Ensemble mean precipitation, wind speed and minimum temperature (hereafter, “temperature”) data of E-OBS daily gridded data (V28.0e) for Europe^34^ during 1950–2022 at 0.25-degree horizontal resolution have been used to study the impact of AR uncertainty on meteorological extremes and variability over Europe. While precipitation and temperature are available from 1950, wind speed data are available from 1980.

In this work, we utilised the TECA-BARD, developed by O’Brien et al.^29^ in 2020, to explore the uncertainty explained by the magnitude of ARP. These ARPs are computed by aggregating binary indicators of AR detection from each of the 1024 detectors in the statistical machine learning model at a given time step. The discrepancies in detections per time step among the 1024 detectors in the model cause variability in the spatial footprints of ARP magnitudes over a location, indicating uncertainty. We examined the impact of these mapping structural uncertainties on the AR characteristics over land and associated anomalies and extremes, including wet, windy, and warm/cold conditions.

The TECA pipeline that makes up the TECA-BARD application can be found in Fig. 7 of O’Brien et al.^29^. The TECA-BARD algorithm takes in NetCDF input that includes specific humidity and wind data and produces an output of AR parameters at a given time step, as described in Table 2. The development of the TECA-BARD and implementation details are provided in O’Brien et al.^29^. The AR parameters are obtained from TECA-BARD from the background field IVT, which can be computed at a given time step using pressure level-specific humidity (q in kg kg$^{-1}$), zonal (u in ms$^{-1}$), and meridional wind (v in ms$^{-1}$) components as per the equations 1, 2, and 3^35^.

1 $$\begin{aligned} \text {IVT} = \sqrt{(\text {IVT}_u)^2 + (\text {IVT}_v)^2} \end{aligned}$$

where the zonal component of IVT (IVT$_u$) is given by,

2 $$\begin{aligned} \text {IVT}_u = \frac{1}{g} \int _{p_s}^{p_t} q \, u \, dp \end{aligned}$$

and the meridional component of IVT (IVT$_v$)

3 $$\begin{aligned} \text {IVT}_v = \frac{1}{g} \int _{p_s}^{p_t} q \, v \, dp \end{aligned}$$

where g is the acceleration due to gravity (ms$^{-2}$), P$_s$ and P$_t$ are the pressure levels (in pascals) at the surface and the top (300 hPa) of the atmosphere, respectively. We obtained the model results at 6-hourly time steps from 1940 to 2022, totalling data over 121,264 time steps (Table 2). Daily means (average of 4-time steps) of ARs (IVTs) are computed^35^ to match the temporal resolution of precipitation, wind speed, and temperature for further analysis. Although the ERA5 data is available at 1-h intervals, making it advantageous for short-term analysis and forecasting over a small area or location, it is computationally expensive when used for 83 years over a larger area and may not significantly impact the results.

**Table 2:** List of parameters in the NetCDF file output from TECA-BARD.

S. No.	Parameter	Description
1	AR_PROBABILITY (ARP)	The posterior probability of the presence of an AR
2	IVT	The L2 norm of (ivt_u_, ivt_v_) shown in Eq. (1)
3	IVT$_U$	The longitudinal component of IVT
4	IVT$_V$	The latitudinal component of IVT
5	AR_BINARY_TAG (ARBT)	Binary indicator of AR; derived by thresholding ARP $\ge$ 0.67
6	AR_COUNT	Number of detections for the parameter table row at the same index in parameter_table_row
7	PARAMETER_TABLE_ROW	The parameter table row corresponding to the value at the same index in ar_count

## Results

### Mapping Euro-Atlantic ARs using TECA-BARD

**Fig. 1:**
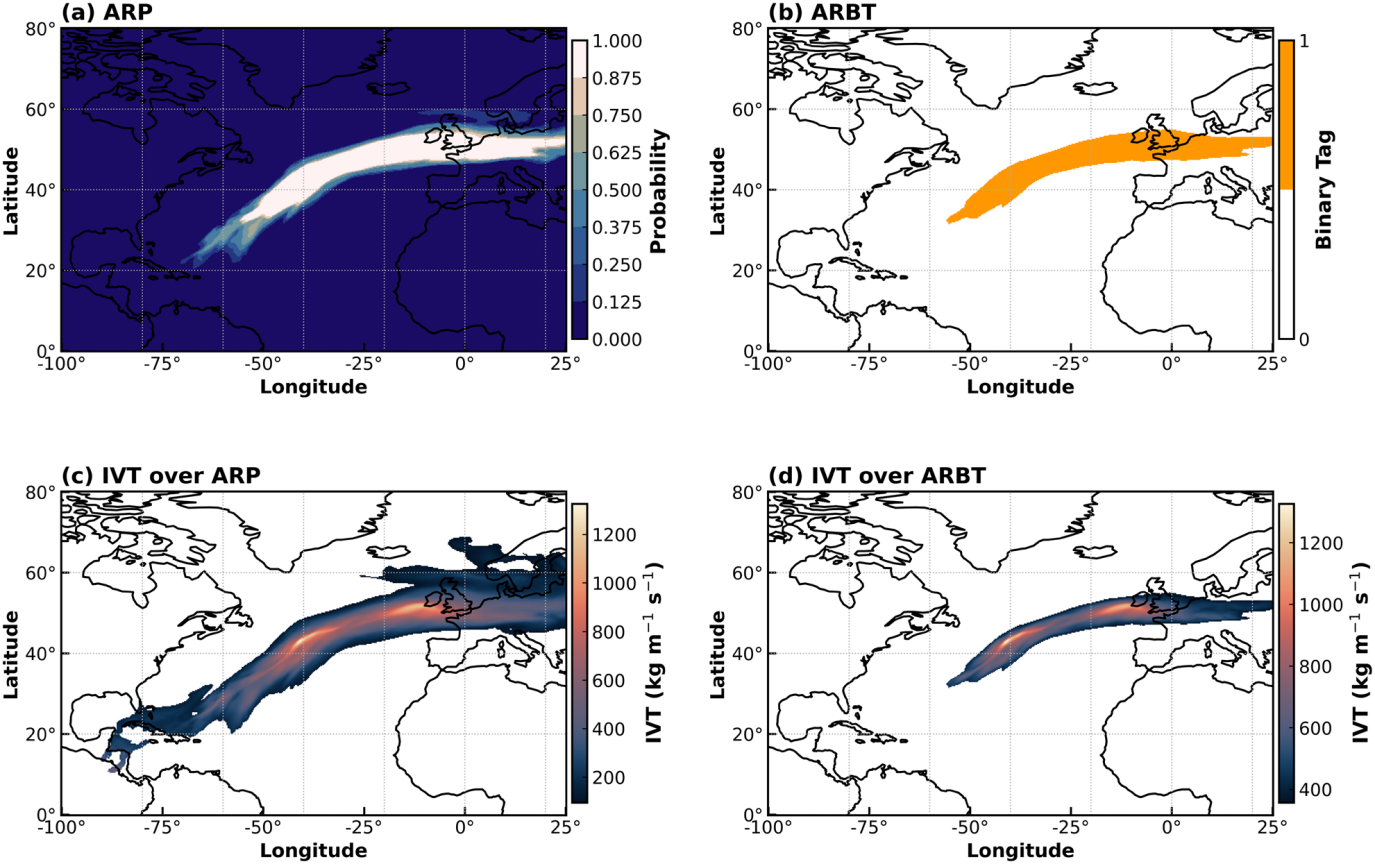
The output of TECA-BARD on 23 February 2020, 00:00 h: (**a**) a full spectrum of ARP, (**b**) an AR binary tag (ARBT) with ARP $\ge$ 0.67, (**c**) IVT over ARP showing the entire length and width of AR mapped by TECA-BARD (**d**) IVT over ARBT, with reduced uncertainty in the length and width of AR. The figure is generated using Python v3.12.4.

For example, Fig. 1 displays the output of TECA-BARD for an AR on 23 February 2020, covering the Euro-Atlantic region. A strong westerly flow over northwest Europe dominated February 2020, and the strong cyclonic activity over the North Atlantic was associated with a strong positive phase of the North Atlantic Oscillation (NAO), leading to several ARs embedded in severe cyclones that affected the region during this period. Europe experienced ARs in connection with storm Ciara (8–9 February) and Dennis (15–16 February), as well as in further weather systems on 22-23 and 28–29 February. The ARs brought strong winds and heavy rainfall, resulting in extremely high levels of precipitation over the month. Monthly precipitation records were broken in England, Wales, Denmark, and parts of southern Sweden, resulting in flooding.

**Fig. 2:**
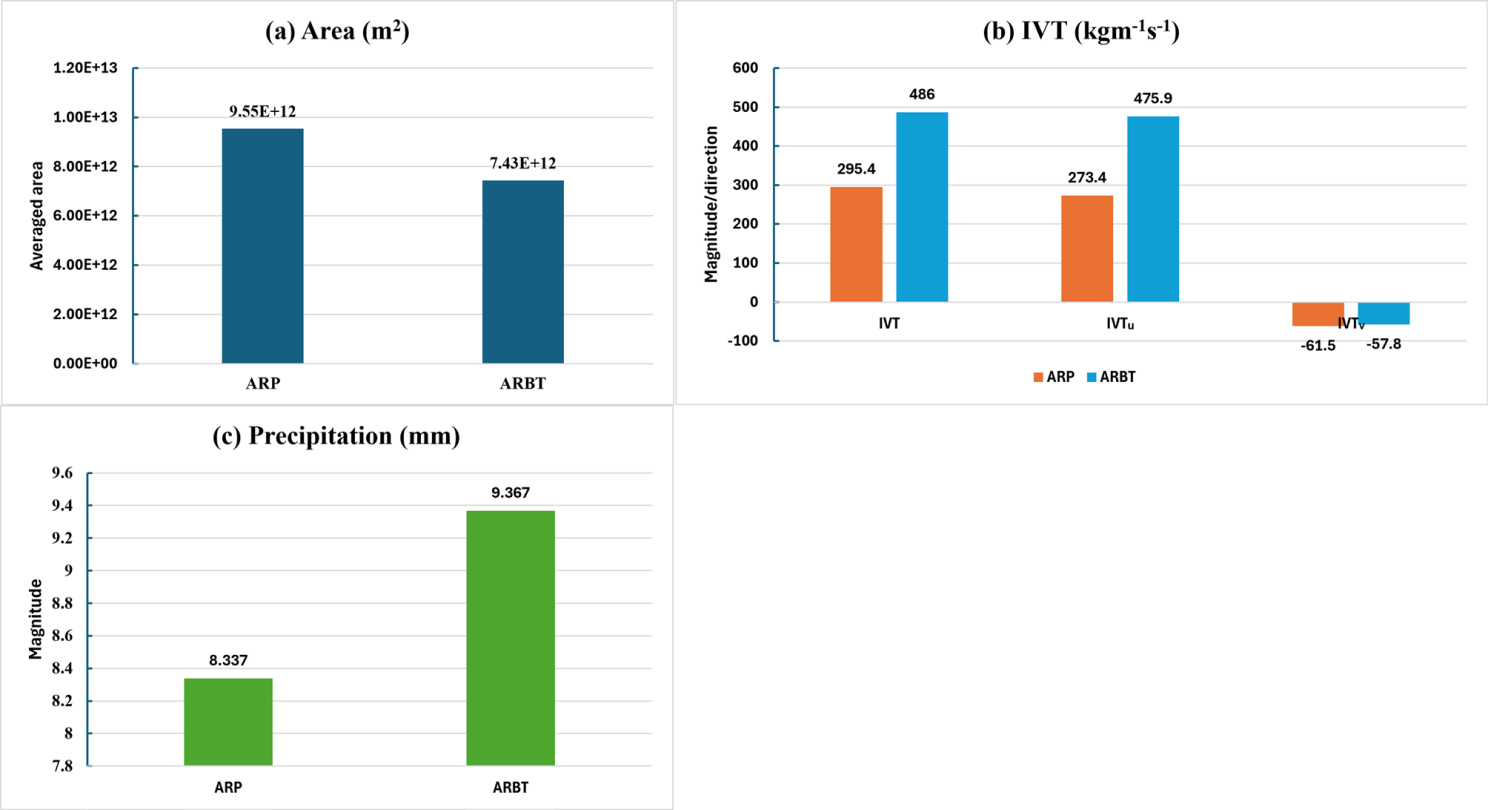
Uncertainties in AR-induced impacts due to selected probabilities for the event on 23 February 2020, 00:00 h in (**a**) Area covered by AR, (**b**) IVT and its components from AR and (**c**) precipitation from AR.

There is a large difference in the dimensions (length and width) of the AR mapped by the ARP (Fig. 1a) and the corresponding binary tag (Fig. 1b), which is ARP $\ge$ 0.67. While IVT over the ARPs ranges from 200 to 1000 kgm^-1^s^-1^ (Fig. 1c), “High” confidence ARs correspond to higher IVT values ($\ge$ 500 kgm^-1^s^-1^) concentrated over a narrow region (Fig. 1d). Thus, “high” confidence ARs can better represent large IVT areas or areas with a larger impact potential during landfalling. This also indicates that location-specific ARPs are heavily reliant on IVT magnitudes. When the minimum area and tropical filter attributes are met, the AR detection parameter sets show good agreement in detecting the AR pixels (areas/grids) when higher IVTs are present. As a result, higher IVTs lead to “high” confidence ARs with higher ARP values and affect the length and width of the ARs mapped by TECA-BARD. Figure 2 shows the difference in magnitudes of the area covered by ARP and ARBT, their area-averaged IVTs and IVT components, and the associated precipitation for the event on 23 February 2020, 00:00 h. High IVT of “high” confidence ARs concentrated over a narrow region (Fig. 2a) due to strong zonal flow (IVT$_U$) led to 1 mm higher area-averaged precipitation than ARP (Fig. 2b,c). The uncertainty in the total area covered by the AR due to the narrow lateral boundaries of the ARBT is approximately 2.12 $\times$ 10^12^ square meters for the event studied. A significant portion of the difference in IVT between the ARP and ARBT, which is 190 kg m^-1^s^-1^, is attributable to the difference in zonal flow, measured at 203 kg m^-1^ s^-1^. In contrast, the meridional component (IVT$_V$) between ARP and ARBT is relatively small ($\approx$ 4 kg m^-1^ s^-1^).

### Spatiotemporal changes

**Fig. 3:**
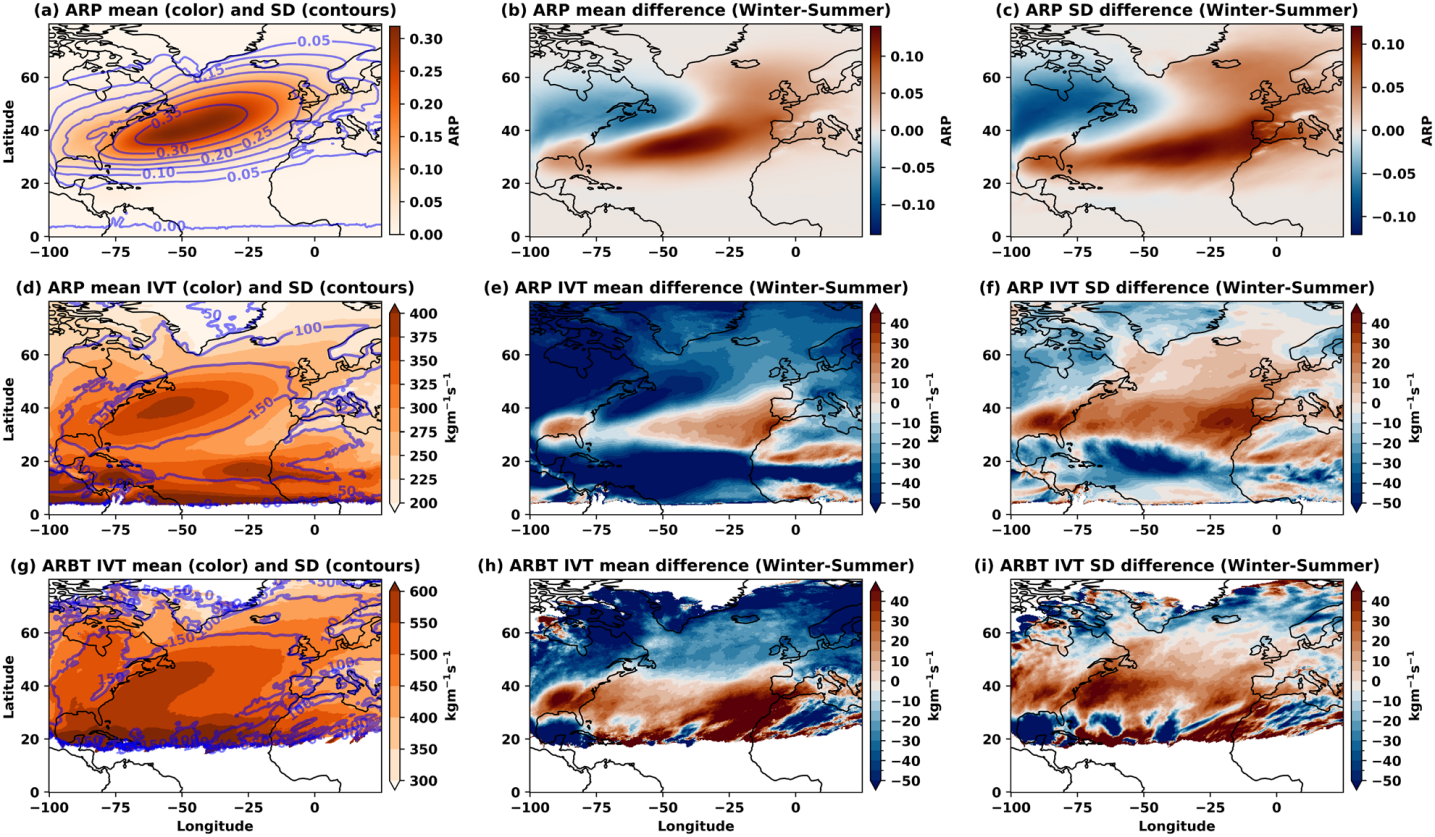
Dependence of IVT variability on the ARP and ARBT over the Euro-Atlantic region during 1940–2022 (**a**) Mean and standard deviation of ARP, (**b**) Difference of ARP between winter and summer half-years, (**c**) Difference of standard deviation between winter and summer half-years. Corresponding IVT mean and standard deviations are shown for ARP in the 2^nd^ row (**d**–**f**) and for ARBT in the 3^rd^ row (**g**–**i**). The figure is generated using Python v3.12.4.

The spatiotemporal analysis of ARP and associated IVT variability over the Euro-Atlantic region reveals fundamental insights into ARs’ behaviour and detection uncertainty (Fig. 3). The mean ARP and standard deviation concentrate over the eastern and central North Atlantic, extending northeast along the storm track toward Western Europe (Fig. 3a). This spatial pattern reflects the climatological pathway of moisture transport, with higher IVT values channelled toward Western Europe through the predominant storm track. The pronounced dipole pattern in winter-summer ARP differences (Fig. 3b,c) demonstrates that AR activity increases by $\approx$ 10% in the southeastern Atlantic during winter while decreasing by $\approx$ 10% over the eastern United States. This seasonal redistribution suggests that the North Atlantic storm track undergoes significant meridional shifts, with winter intensification associated with enhanced baroclinic instability and stronger temperature gradients. The comparison between ARP (starting at 5$^{\circ }$N) and ARBT (starting at 20$^{\circ }$N) reveals critical implications for the detection methodology. ARP captures tropical moisture contributions that ARBT filters out, suggesting that lower detection thresholds may be necessary to fully characterise moisture transport from tropical convection. This methodological difference has direct implications for understanding AR genesis and evolution. The higher IVT mean and standard deviation of ARBT along the Gulf Stream (Fig. 3g–i) quantify the ocean’s role as a moisture source for AR development. The enhanced winter air–sea flux exchange over warm SSTs creates optimal conditions for “high confidence ARs,” explaining the temporal variability in ARP values and supporting the physical basis for seasonal AR intensity changes^36–38^.

The larger differences in ARP standard deviations compared to means (extending from southwestern Europe to Scandinavia) indicate that detection uncertainty varies spatially and seasonally. This finding suggests that AR detection algorithms may perform differently across regions and seasons, with implications for both climatological studies and operational forecasting. While ocean regions show substantial variability in ARP and IVT, the smaller but significant uncertainty over land areas could propagate into errors in AR dimensional characteristics and IVT calculations. This land-ocean contrast highlights the need for region-specific validation approaches in AR detection schemes. The TECA-BARD model’s lack of seasonal disaggregation in parameter selection, while maintaining consistent global performance (± 1 seasonal range), suggests that the observed seasonal patterns reflect genuine atmospheric variability rather than detection artefacts^29^. However, the substantial seasonal differences in ARP magnitude indicate that future detection schemes might benefit from seasonally-adjusted thresholds to optimise performance across different atmospheric regimes. These findings collectively demonstrate that AR detection uncertainty is not merely a technical issue but reflects fundamental aspects of atmospheric variability that must be considered in both climatological analyses and operational applications.

### Uncertainty in AR frequency

**Fig. 4:**
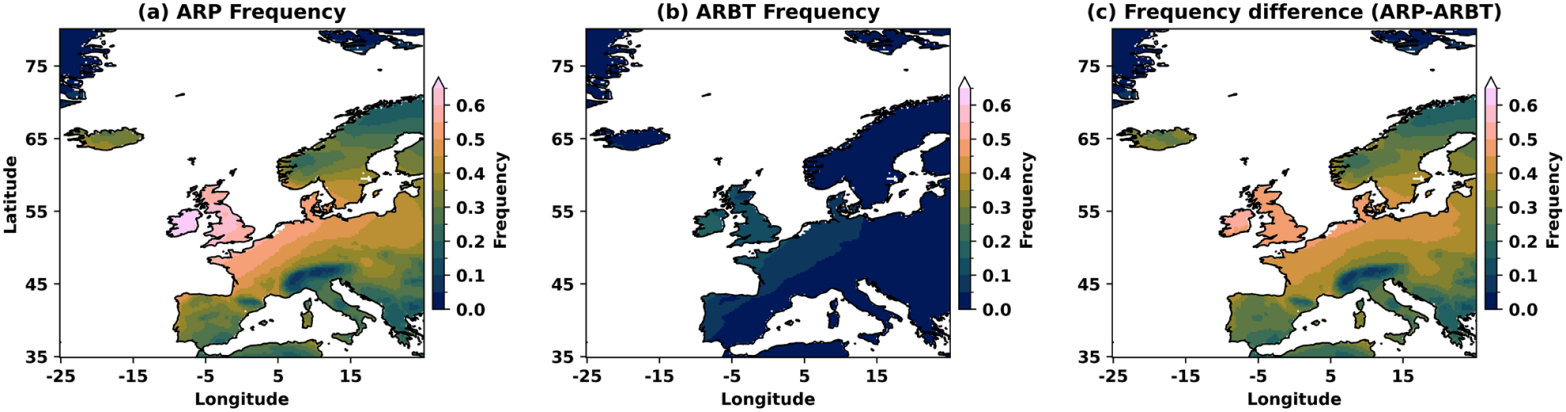
AR frequencies of occurrence calculated over Europe using 6-hourly TECA-BARD output data (121264-time steps) during 1940–2022 from (**a**) ARP, (**b**) ARBT and (**c**) the difference between ARP and ARBT. Frequency is calculated as the ratio of the number of non-zero 6-hourly time steps to the total number of 6-hourly time steps in the study period. The figure is generated using Python v3.12.4.

The AR detections with structural uncertainty show a large difference in the variability of ARs’ spatial frequency over the United Kingdom and Ireland, Europe and Scandinavia (Fig. 4). The high “confidence” (ARBT) ARs are mainly landfalling over the UK and the west coast of Europe, between 45$^{\circ }$ and 60$^{\circ }$ N (Fig. 4b), showing a larger frequency (> 0.1) than other regions. These areas are along the path of the North Atlantic storm track. The frequency of landfalling high-confidence ARs over the inland Iberian Peninsula and Scandinavia is low (< 0.05) and shows the rare occurrence of extreme AR events over these regions. Thus, ARBT cannot account for the higher frequency of occurrence of ARs in southern Europe (the inland Iberian Peninsula) and the Scandinavian Peninsula.

Conversely, the frequency of ARP is high ($\approx$ 0.3) in these regions (Fig. 4a), along with coastal, eastern and inland Europe, where the AR landfalling frequency is high (> 0.4). The UK is exposed to frequent AR landfalls, with the highest frequency (> 0.7), and the Alps shows the lowest. The regions along the North Atlantic storm track, including eastern and inland Europe and southern Sweden, show a larger frequency difference between ARP and ARBT (Fig. 4c). The physical features, orography, have a significant impact on the frequency of ARs across Europe, the UK and Scandinavia. The leeward side of high mountains, such as those found in the central plateau of the Iberian Peninsula and the Alps, creates AR “shadow” regions with low ARPs and almost no ARBTs. Thus, the structural uncertainties in ARs (selection of ARP/ARBT) can bring a large difference in landfalling frequencies and may result in uncertainties in associated wet, windy and warm/cold anomalies and extremes. To explore these uncertainties in wet, windy and warm/cold anomalies and extremes based on the ARP selected, we divide the ARP into 10 equal parts with an increase of 0.1, called “deciles”, and explored the impact of AR footprints associated with these structural uncertainty (deciles) on AR characteristics and wet, windy and warm/cold anomalies and extremes in the following sections.

### Impact on AR characteristics and meteorological variables over land

**Fig. 5:**
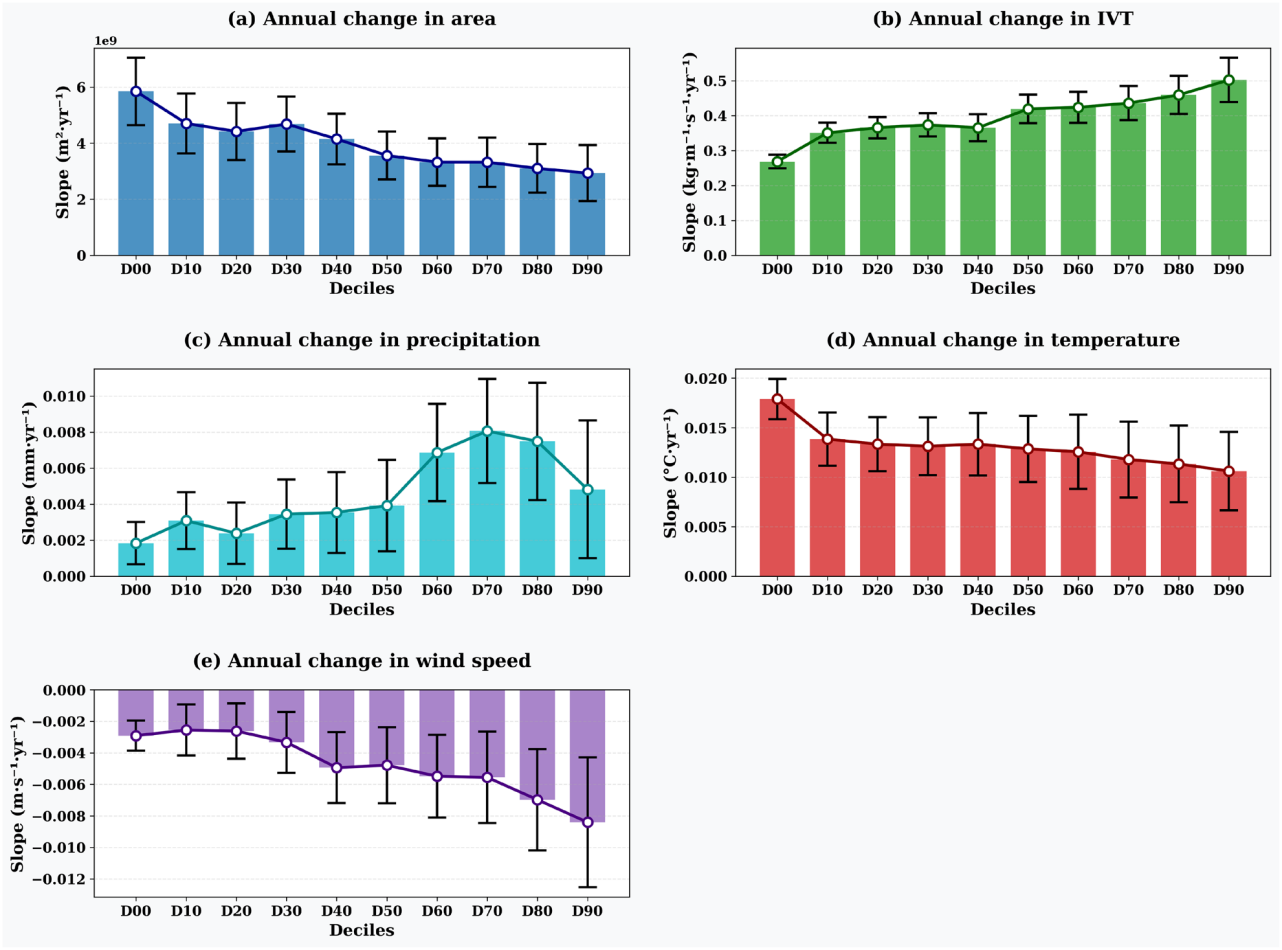
The impact of ARP deciles on the annual trends of (**a**) AR occurrence area, (**b**) IVT, (**c**) precipitation, (**d**) temperature, and (**e**) wind speed during landfalling over land. The figure is generated using Python v3.12.4.

**Fig. 6:**
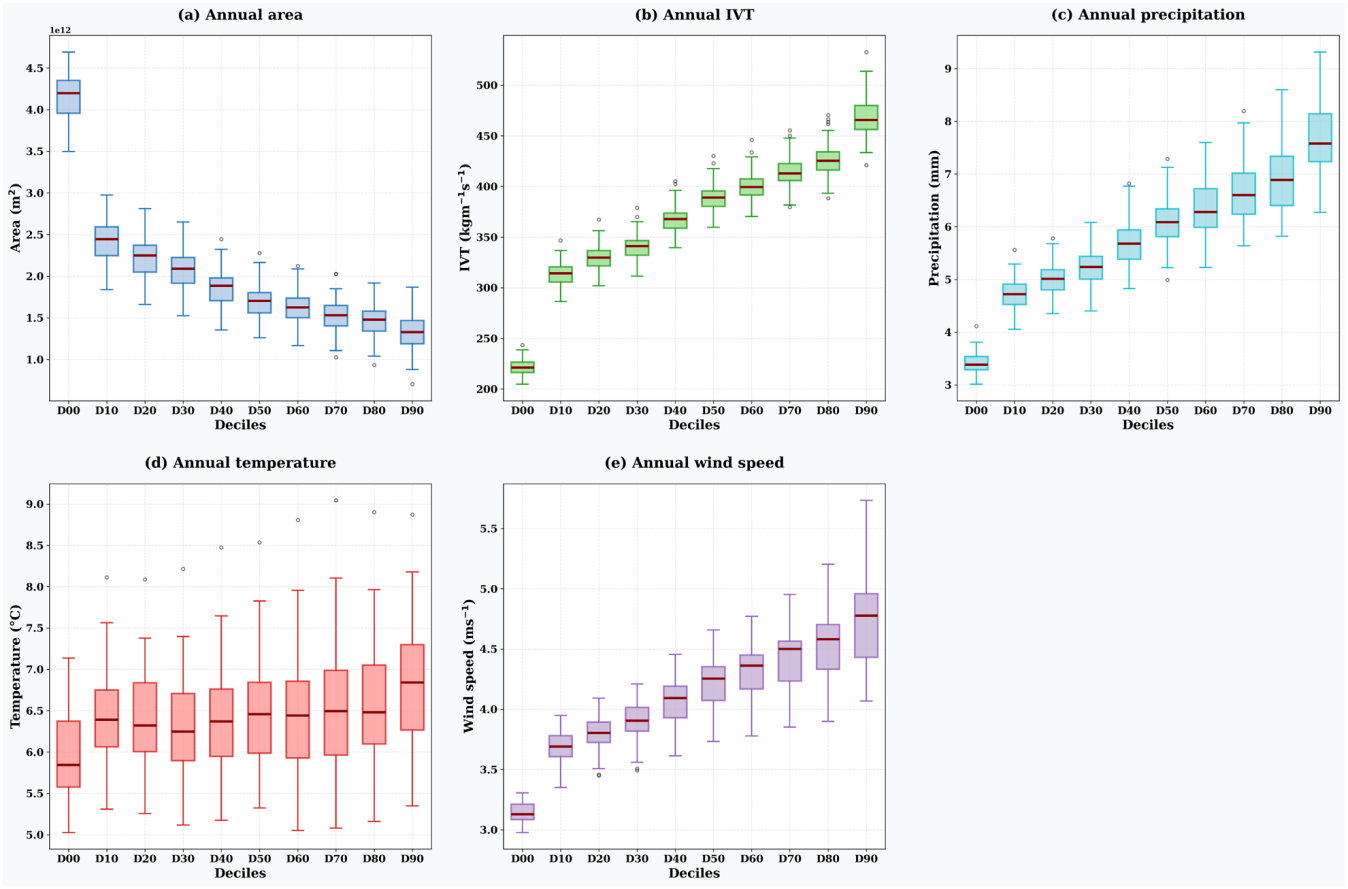
Whisker-box plot showing the spread of annual area mean of AR characteristics during landfall based on selected deciles for (**a**) Landfalling area, (**b**) IVT, (**c**) precipitation, (**d**) temperature, and (**e**) wind speed. The figure is generated using Python v3.12.4.

Figures 5 and 6 illustrate the impact of structural uncertainty (represented by the ARP deciles) on the annual AR characteristics and the associated meteorological parameters over land. In addition to the default 0.67 threshold used for ARBT, we explore threshold choices in the range [0.0, 0.1 ... 0.9] and refer to these as D00, D10, ... D90. This will narrow the area represented by the ARs by increasing the “confidence” in AR detection (and reducing uncertainty), as shown in Figs. 5a and 6a. Figure 5 shows the annual temporal trends (regression slopes concerning time in years) for various AR-related parameters across different ARP deciles. The y-axis represents the slope of the linear regression fitted to the annual time series data, with units indicating the rate of change per year. The annual mean AR area (Fig. 5a) shows temporal trends ranging from approximately 6 $\times$ 10^9^ m^2^ y^-1^ for D00 to 3 $\times$ 10^9^ m^2^ y^-1^ for D90, indicating that AR occurrence/impact areas are increasing over time irrespective of the selected decile, with the rate of increase being halved when comparing the lowest to highest decile thresholds. The temporal trend coefficients show a 2-fold change between D00 and D40 (from $\approx$ 6 $\times$ 10^9^ to $\approx$ 4 $\times$ 10^9^ m^2^ y^-1^) and vary less from D50 to D90 ($\approx$ 3.5 $\times$ 10^9^ to $\approx$ 3 $\times$ 10^9^ m^2^ y^-1^), showing reduced sensitivity of ARP selection on land area trends at higher deciles.

The IVT temporal trends from selected ARP deciles show an opposite pattern (Fig. 5b), with smaller annual trend coefficients at D00 ($\approx$ 0.27 kg m^-1^ s^-1^ y^-1^) increasing to larger values at D90 ($\approx$ 0.48 kg m^-1^ s^-1^ y^-1^). The increase in ARP deciles leads to higher “confidence” in AR regions with stronger positive IVT trends over time. The annual temporal trend coefficients show minimal change between D10 to D40 ($\approx$ 0.35–0.38 kg m^-1^ s^-1^ y^-1^) and D50–D80 ($\approx$ 0.40–0.45 kg m^-1^ s^-1^ y^-1^), making the ARP selection less significant among these intermediate deciles. However, these similarities in area and IVT temporal patterns do not correspond to precipitation trends (Fig. 5c). Selected deciles greatly affect the annual precipitation temporal trends, with ARPs above D40 showing large variability in annual precipitation trend coefficients. D40 and D50 show similar annual precipitation trend increases ($\approx$ 0.0035 mm y^-1^), but the annual temporal trends for D60 and above are substantially larger ($\approx$ 0.008 mm y^-1^ for D70) compared to lower deciles. The annual temporal trends in temperature show minimal change, with selected deciles of D10 and above exhibiting relatively stable trend coefficients of around 0.012–0.014 $^{\circ }$C y^-1^ (Fig. 5d). Similarly, as the AR area narrows with increasing ARP deciles, the corresponding annual temporal trends in area-averaged mean wind speeds show increasingly negative values (more negative trends), despite the concentration of large IVTs in the higher deciles. The selection of D00 to D30 shows relatively constant negative temporal trends ($\approx$ $-0.002$ to $-0.003$ m s^-1^ y^-1^), while higher deciles show more pronounced negative trends (D90: $\approx$ $-0.008$ m s^-1^ y^-1^) (Fig. 5e).

Figure 6 presents the distributional characteristics (box plots showing quartiles, medians, and outliers) of AR-related parameters across different ARP deciles, representing the statistical distribution of area-averaged values within individual AR events during the study period. Figure 6a shows the distribution of individual AR areas at landfall, with median values decreasing from approximately 4.2 $\times$ 10^12^ m^2^ for D00 to 1.4 $\times$ 10^12^ m^2^ for D90, confirming the narrowing of AR areas with increasing ARP thresholds. Figure 6b displays the distribution of area-averaged IVT values within individual ARs, showing median values increasing from $\approx$ 220 kg m^-1^ s^-1^ for D00 to $\approx$ 470 kg m^-1^ s^-1^ for D90, indicating that higher ARP deciles capture ARs with stronger moisture transport intensities. Figure 6c illustrates the distribution of area-averaged precipitation within ARs, with median values increasing from $\approx$ 3.5 mm for D00 to $\approx$ 7.5 mm for D90. The increasing spread and magnitude of precipitation distributions at higher deciles (particularly D60–D90) correspond to the large variability observed in precipitation temporal trends. Figure 6d shows the distribution of area-averaged temperatures within ARs, with relatively stable median values ($\approx$ 6.5–7.0 $^{\circ }$C) across most deciles, consistent with the minimal changes in temperature temporal trends. Figure 6e presents the distribution of area-averaged wind speeds within ARs, showing median values increasing from $\approx$ 3.1 m s^-1^ for D00 to $\approx$ 4.8 m s^-1^ for D90.

### Impact on compound and concurrent meteorological extremes

Compound extremes are events in which multiple types of extremes co-occur or in close succession, leading to enhanced impacts due to their interactions. These events involve combinations of multiple climate or weather variables (e.g., temperature and precipitation, wind and sea level) or spatial dependencies (e.g., multiple regions experiencing extremes at once), or may include cascading effects where one extreme triggers another (e.g., heavy rainfall leading to landslides). The combined occurrence of extremes can exacerbate risks and consequences for ecosystems, infrastructure, and human systems^39^. Concurrent extremes refer to the simultaneous occurrence of extreme weather or climate conditions at different locations or within multiple variables in the same location. Unlike compound extremes, which emphasise the inter-variable interactions or cascading effects, concurrent extremes typically involve simultaneous extreme events in separate areas or simultaneous extremity in multiple variables without direct interaction^40^.

Successive AR events, also known as AR families, can result in compound hydrological extremes due to limited recovery time between periods of landfall^41,42^. This can lead to enhanced hydrological and socioeconomic impacts, particularly over the coasts of Europe, the UK, the United States, and Norway^8,11,43–46^. However, there is a lot of uncertainty around the occurrence and magnitude of compound meteorological extremes when individual AR landfalls^8,47^, as the characteristics of individual ARs depend on the mapping techniques/algorithms and data used^48^. This uncertainty is further compounded by the qualitative segregation of the different types of ARs (flavours) and their large-scale flow patterns^41,49–51^.

**Fig. 7:**
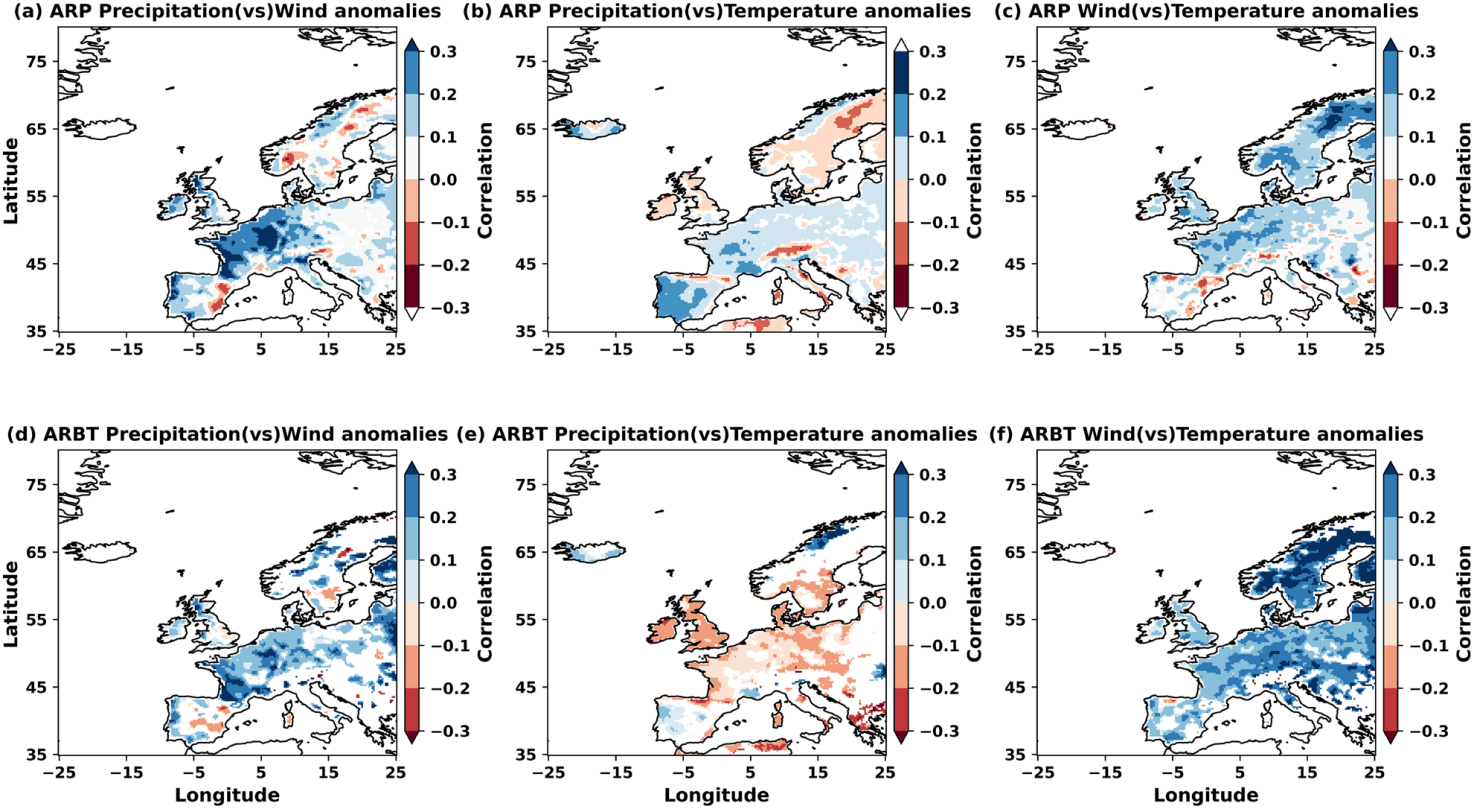
Compoundness and concurrency among anomalies of meteorological variables during AR landfall based on ARP (row 1: **a**–**c**) and ARBT (row 2: **d**–**f**). All correlations shown are significant at 95%. The figure is generated using Python v3.12.4.

To better understand the compound and concurrent meteorological variables during individual AR landfall, the simultaneous correlations among their anomalies are examined using daily data. As it is increasingly complex to show how all the ARPs can affect this compoundness and concurrency among AR-induced meteorological variables, the analysis was limited to ARP and ARBT. Figure 7 illustrates the correlations among precipitation, wind speeds, and temperature during AR landfall, considering ARP and ARBT. These daily anomalies of meteorological variables are computed from the daily climatologies of the data in the study period. AR landfall caused increased wind speeds and precipitation anomalies along coastal Europe, the UK, and northern Norway, with larger (Fig. 7a) and lower (Fig. 7d) structural uncertainty cases, with minor changes between the two selections made. However, inland (Eastern Europe) areas lacking terrain features show lower correlations between anomalous wind speeds and precipitation when structural uncertainty in AR mapping increases. This variability can be attributed to vertical uplift and condensation factors that ultimately determine the efficiency with which IVT is converted to precipitation, including the orientation of winds relative to terrain. Thus, the impact of wind speeds and directions during AR landfall on precipitation patterns depends on the region’s topography and the storm’s orientation^49^, which are sensitive to the AR dimensions/size.

Similarly, reduced uncertainty in AR mapping shows a negative correlation between associated precipitation anomalies and temperature (Fig. 7e). Enhanced AR-induced precipitation can lower the temperatures in the UK and Europe, or vice versa. This relationship breaks down in the Iberian Peninsula (south) and coastal Norway (north), which shows positive correlations. In contrast, increased uncertainty in AR mapping (larger AR lateral boundaries) led to a positive correlation between these variables over most parts of Europe, including coastal Norway (Fig. 7b). Also, negative correlations over the UK and Scandinavia have dipped in magnitude with increased uncertainty in AR mapping. Further, ARs are typically associated with a “low-level” jet causing strong winds that can transport warm, moist air from lower latitudes to higher latitudes. These “windy” ARs cause warm air advection. They can increase temperatures in the landfalling regions (Fig. 7c,f), leading to positive correlations with wind speeds over coastal Europe, the UK and Scandinavia. Correlations are stronger over areas with less attenuation from the AR winds, such as Scandinavia and along the great European plains extending towards Eastern Europe. The areas with minimal uncertainty (larger IVTs) led to more significant warming over land, resulting in higher correlations in the case of “high” confidence ARs than in ARP.

Moreover, no simultaneous significant correlations were found between the precipitation and wind extremes (Supplementary Figures S1a and S1d) from ARBT. Provided favourable conditions, moderate ARs can cause extreme precipitation, coupled with strong winds, over parts of Spain, France, and central Europe. Moderate correlations are found between extreme precipitation and temperature during low and moderate ARs, with larger uncertainty over Eastern Europe and Scandinavia (Supplementary Figure S1b and S1e). A strong and “windy” AR can cool the surface temperatures along the UK and coastal Europe, leading to mild negative correlations between extreme winds and temperatures. On the other hand, winds from moderately intense ARs with lower ARPs (higher uncertainty) can cause negative correlations over Scandinavia and Eastern Europe (Supplementary Figure S1c and S1f). Intense/extreme ARs are largely characterised by either wet or windy and warm conditions over large parts of the UK, Scandinavia and Europe. While extreme precipitation from ARs with larger uncertainty led to temperatures increasing, events associated with extreme winds led to colder temperatures over land.

**Fig. 8:**
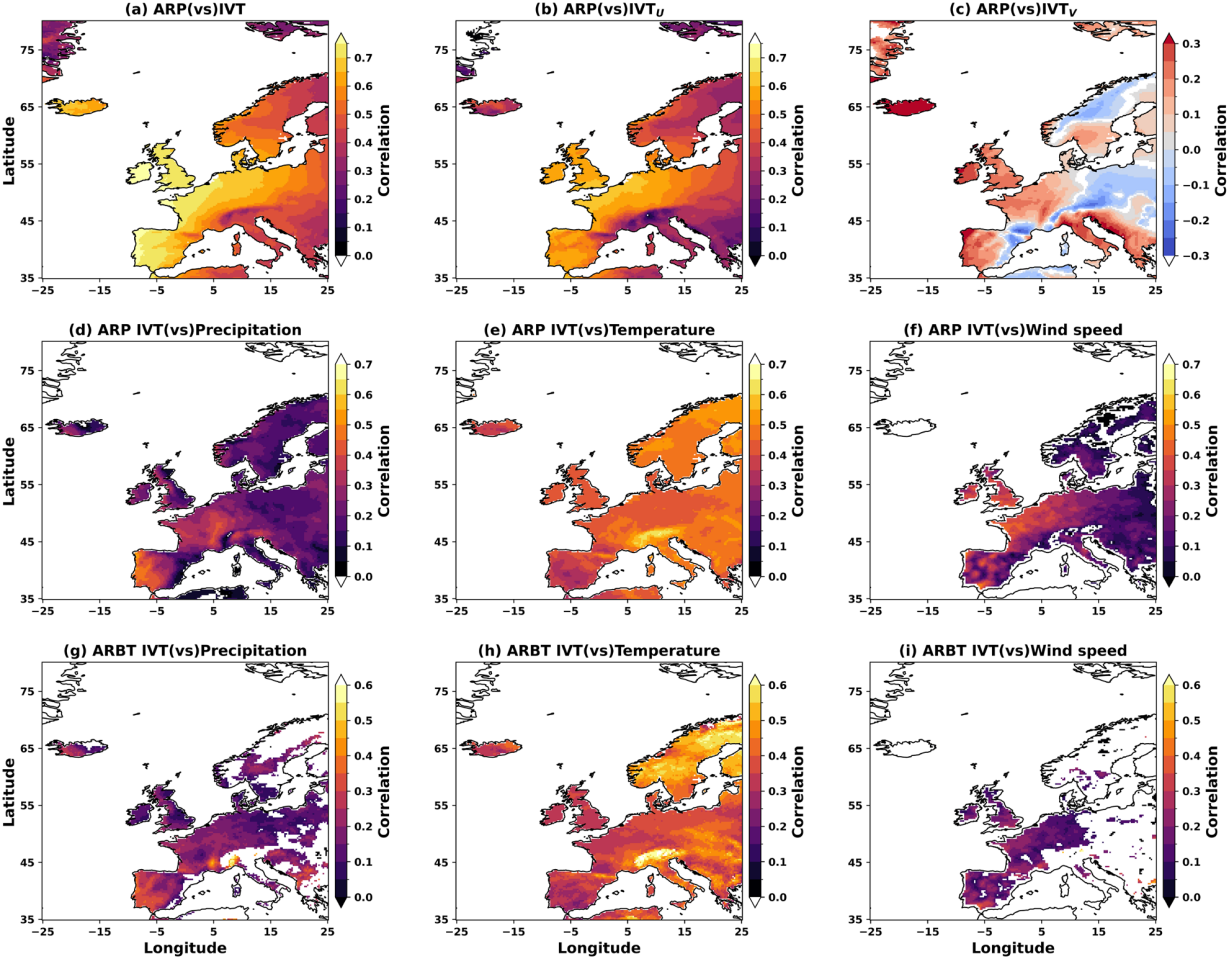
The spatial variability of correlations for ARP and (**a**) IVT, (**b**) IVT$_U$, and (**c**) IVT$_V$. Similarly, correlations for precipitation, temperature, and wind speeds with corresponding IVTs from ARP (**d**–**f**) and ARBT (**g**–**i**) are shown in the 2^nd^ and 3^rd^ rows, respectively. All correlations shown are significant at 95%. The figure is generated using Python v3.12.4.

To investigate the connection between the landfalling ARs and the effects of associated IVTs on wet, windy, and warm/cold weather conditions, we further analysed their variability with ARP and ARBT. The correlation analysis reveals that a higher AR frequency is robustly associated with higher IVT, precipitation, temperature, and wind speeds across most of Europe. This relationship remains consistent regardless of the ARP threshold (Fig. 8). However, the strength of these correlations varies systematically with ARP threshold, revealing how structural uncertainty in AR mapping affects the quantification of AR-induced meteorological phenomena. AR frequency positively correlates with IVT magnitude, precipitation, temperature, and wind speed across Europe, with all correlations shown being significant at a 95% confidence. This robust relationship holds for both ARP and ARBT detection methods, again establishing that the fundamental AR-climate relationship is not an artefact of detection methodology. The spatial patterns show the strongest correlations over the UK, Western Europe, and the Iberian Peninsula, regions in the primary AR moisture transport corridor. ARP consistently produces stronger correlations than ARBT across most meteorological variables and regions. This systematic difference indicates that including moderate-intensity ARs (captured by ARP but filtered out by ARBT) strengthens the statistical relationship between AR frequency and meteorological impacts. The zonal IVT (IVT$_U$) exhibits robust correlations with ARP across Europe (Fig. 8b). In contrast, the meridional transport (IVT$_V$) influences are more regionally constrained, with negative correlations over Eastern Europe and Scandinavia reflecting southward moisture transport patterns (Fig. 8c). Thus, the orientation of the ARs along with the associated intensity of extra-tropical storms could impact the AR strength and guide ARP^52–56^.

While the positive AR-meteorological relationships are robust, the magnitude of correlations varies by meteorological variable. Precipitation correlations (Fig. 8d,g) show the largest differences between ARP and ARBT, with ARP extending significant correlations into Eastern Europe, where moderate-intensity ARs contribute to precipitation variability. Temperature correlations (Fig. 8e,h) reveal regional sensitivity, particularly over Scandinavia, where the detection approach substantially affects the strength of AR-temperature relationships. Wind speed correlations (Fig. 8f,i) demonstrate that ARP captures broader spatial coverage while ARBT focuses on coastal regions with the most intense AR impacts. The systematic difference in correlation strength between ARP and ARBT reveals that AR impact assessments are inherently sensitive to detection methodology, with the choice of detection approach potentially leading to 20–40% differences in estimated AR contributions to regional meteorological variability. This sensitivity varies spatially, with Scandinavia and Eastern Europe showing the largest differences between detection approaches, indicating these regions are particularly vulnerable to methodological choices and should be prioritised for validation efforts. The robust positive relationships across detection methods provide confidence in the fundamental AR-climate connections. At the same time, the systematic differences in correlation strength highlight the importance of selecting the appropriate detection approach. For comprehensive impact assessments, the choice between broader coverage (ARP) and an intensity focus (ARBT) should be guided by the specific application. Broad coverage may be preferable for regional climate studies, while an intensity focus may be more suitable for analysing extreme events over the region.

**Fig. 9:**
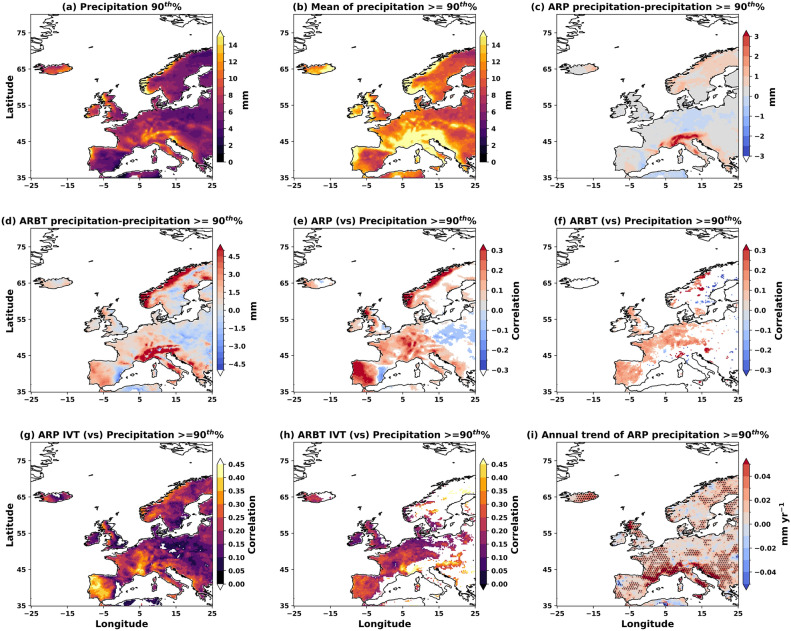
The impact of ARP, ARBT and associated IVTs on extreme daily precipitation over Europe. (**a**) The magnitude of 90^th^% precipitation, (**b**) mean precipitation $\ge$ 90^th^%, (**c**) mean extreme precipitation with ARP and (**d**) with ARBT. The correlations (significant at 95%) of extreme precipitation with (**e**) ARP, (**f**) ARBT, and associated IVTs (**g**, **h**), respectively, and (**i**) the annual trend in extreme precipitation linked to ARP.

**Fig. 10:**
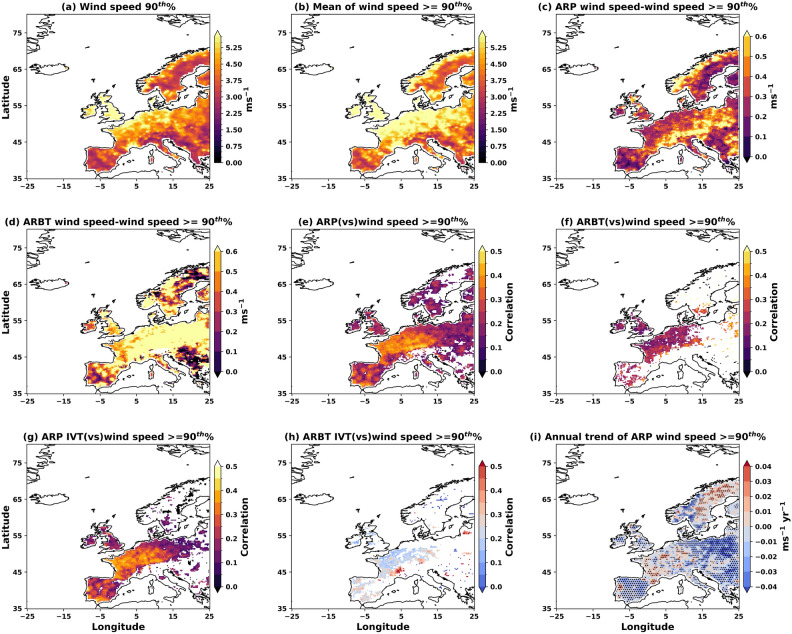
Impact of ARs on extreme wind speeds (daily mean) over Europe. (**a**) 90^th^% wind speeds, (**b**) mean wind speeds $\ge$ 90^th^%, (**c**) the mean wind speeds $\ge$ 90^th^% with ARP, and (**d**) with ARBT. The correlations (significant at 95%) of extreme wind speeds with (**e**) ARP, (**f**) ARBT, and associated IVTs (**g**, **h**), respectively, and (**i**) the annual trend in extreme wind speeds from ARP. All correlations shown are significant at 95%. The figure is generated using Python v3.12.4.

**Fig. 11:**
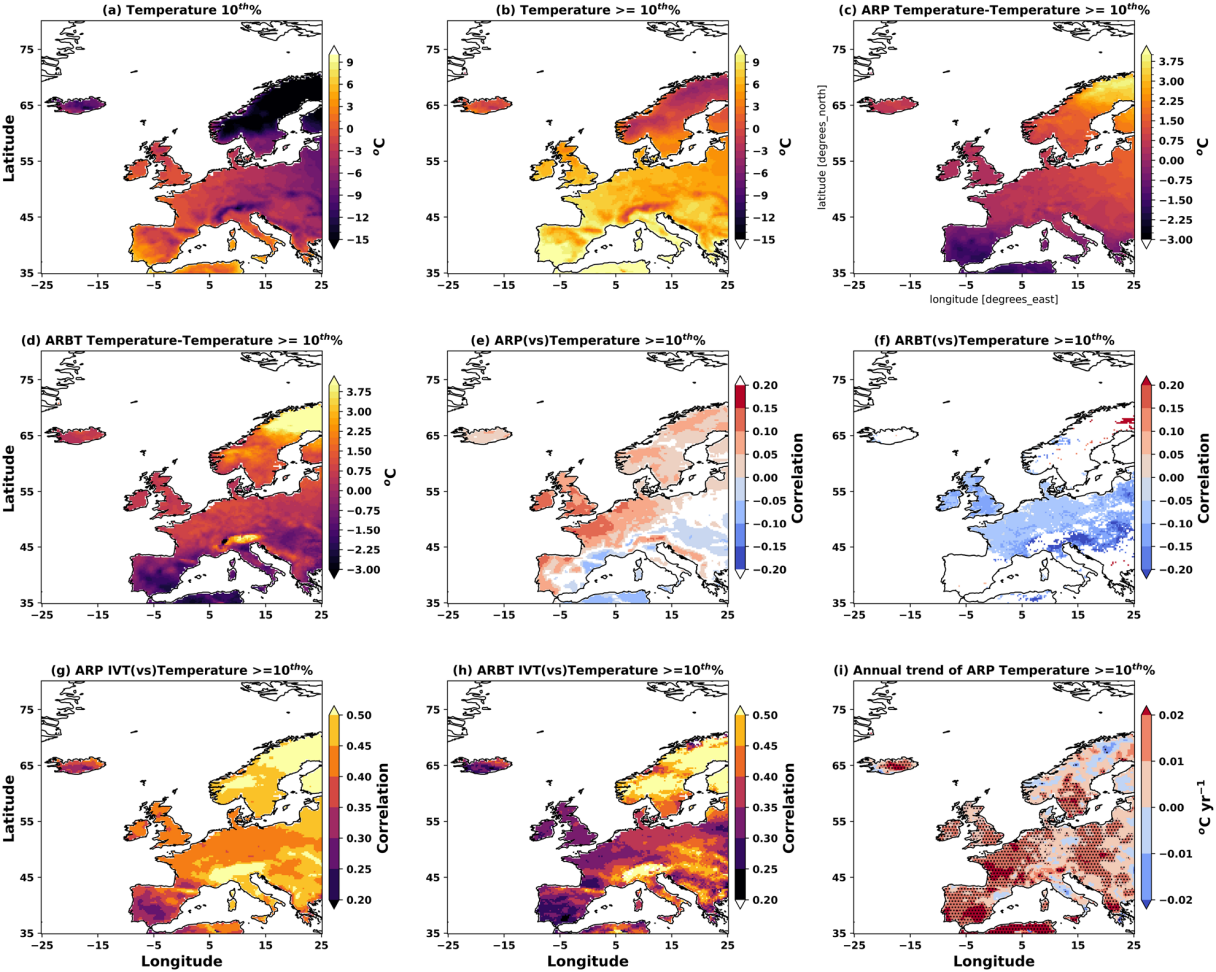
Impact of ARs on daily temperature over Europe. (**a**) 10^th^% temperature, (**b**) mean temperature $\ge$ 10^th^%, (**c**) the mean temperature $\ge$ 90^th^% with ARP, and (**d**) with ARBT. The correlations (significant at 95%) of temperature $\ge$ 10^th^% with (**e**) ARP, (**f**) ARBT, and associated IVTs (**g**, **h**), respectively, and (**i**) the annual trend of temperature $\ge$ 10^th^% linked to ARP. The figure is generated using Python v3.12.4.

The structural uncertainties in ARs significantly affect the spatial variability of daily wet, windy, and warm/cold anomalies and their extremes (Figs. 9, 10, and 11). The analysis of extreme precipitation ($\ge$90^th^ percentile) demonstrates that higher AR frequency is robustly associated with higher extreme precipitation across Europe, with extreme AR precipitation showing increasing trends in most regions (Fig. 9). Both ARP and ARBT show significant positive correlations with extreme precipitation over the UK, coastal Norway, central Europe, and the Iberian Peninsula (Fig. 9e,f). These relationships are strongest in orographically influenced regions where terrain enhances moisture uplift, including the Iberian Peninsula, northwestern UK, Kjolen mountains, and the Alps (Fig. 9a,b). The magnitude of AR-induced precipitation during extreme events varies by detection method, with high-confidence ARs producing precipitation exceeding the 90^th^ percentile by more than 3 mm in key regions (Fig. 9c,d). The correlation patterns using AR-associated IVT (Fig. 9g,h) mirror those using AR frequency, confirming that the IVT intensity of ARs drives the relationship.

ARP captures broader spatial coverage of AR-extreme precipitation relationships compared to ARBT, which focuses on coastal and central European regions with the most intense AR activity. This pattern indicates that moderate-intensity ARs (captured by ARP) also contribute significantly to extreme precipitation under favourable conditions, while high-confidence ARs (ARBT) dominate in regions with the strongest orographic enhancement. Annual trends reveal significant increases in AR-induced extreme precipitation across most European regions, with particularly notable increases over the UK, France, Iberia, Eastern Europe, and parts of Scandinavia at rates of approximately 0.02 mm y^-1^ (Fig. 9i). The Mediterranean region shows the largest increases in annual extreme precipitation associated with ARs, indicating a shift toward more intense AR-related precipitation events in this region. These increasing trends in AR-related extreme precipitation, combined with the strong positive correlations between AR frequency and extreme precipitation, suggest that the intensification of AR activity is contributing to enhanced precipitation extremes across Europe. This has important implications for flood risk assessment, water resource management, and climate adaptation planning, as AR-related extreme precipitation events are becoming both more frequent and more intense. This increase in AR-induced extreme precipitation over parts of the UK, Europe, and Scandinavia is primarily attributed to increased precipitation from “high” confidence ARs (Supplementary Figure S2). There could be multiple reasons for this, such as the increasing AR frequencies and the higher moisture uptake (higher intensities) by ARs due to warmer SSTs over the Mediterranean and Atlantic, which need further investigation.

To examine the role of AR mapping uncertainty on extreme wind speeds (at 10 m), we analyse daily wind speeds exceeding the 90^th^ percentile threshold at each location (Fig. 10a,b). A higher AR frequency is consistently associated with stronger winds across most European regions, and this relationship remains robust across different AR detection thresholds. Coastal regions of Europe, Norway, and the UK exhibit the highest mean wind speeds at the 90^th^ percentile, reaching 5–8 m s^-1^, while inland areas experience lower speeds of less than 4 m s^-1^ (Fig. 10a). The spatial pattern of mean extreme wind speeds shows maximum values of 7–8 m s^-1^ concentrated over coastal Europe, the UK, and Norway (Fig. 10b). AR-induced winds significantly modulate extreme wind speeds across the study domain. Except over coastal Norway and Eastern Europe, AR events enhance extreme wind speeds by up to 0.4 m s^-1^ compared to the climatological mean (Fig. 10c). The influence of AR mapping uncertainty on wind speed patterns becomes evident when comparing different confidence levels: coastal Norway and central Europe experience stronger extreme wind speeds ($\approx$ 1 m s^-1^) during “high” confidence AR landfall events compared to lower confidence events ($\approx$ 0.6 m s^-1^) relative to the 90^th^ percentile baseline (Fig. 10d).

The relationship between AR properties and surface winds varies significantly with AR detection confidence. ARs with higher uncertainty and larger lateral boundaries exhibit strong positive correlations ($\approx$ 0.5) with wind speeds along the Iberian Peninsula and European plains (Fig. 10e), with their associated IVTs accounting for a significant portion of this wind speed variability (Fig. 10g). In contrast, “high” confidence ARs and their IVTs exhibit more moderate correlations of $\approx$ 0.2 (Fig. 10f,h), demonstrating that extreme ARs with larger IVT do not always produce proportionally stronger surface winds, though they are more effective at generating extreme precipitation (Fig. 9). The zonal component of IVT (IVT$_U$) emerges as the primary driver of strong wind-IVT correlations over coastal Europe and the Iberian Peninsula (Supplementary Figure S3a). Conversely, the meridional component (southward-oriented IVT$_V$) enhances northerly flow and reduces surface temperatures, increasing near-surface stability. This stable stratification impedes momentum transfer from landfalling ARs, resulting in weaker surface winds and negative correlations over Eastern Europe and Scandinavia (Supplementary Figure S3b).

AR-induced wind speeds show a systematic decreasing trend across most of the study area (Fig. 10i), with annual reductions of 0.02–0.06 m s^-1^ y^-1^. The largest decreases occur over Eastern Europe, the UK, the Iberian Peninsula, and the Norwegian coast. The cold air advection, driven by enhanced meridional flow, provides a physical mechanism for the observed annual decline in extreme wind speeds from landfalling ARs. Notably, these wind speed changes are primarily driven by “high” confidence or extreme ARs with higher IVTs (Supplementary Figure S3c), indicating that the most intense AR events are undergoing the most significant changes. The key finding is that AR frequency and intensity consistently correlate with enhanced wind speeds across Europe, with this relationship remaining robust regardless of the AR detection threshold applied. However, the strength of this relationship varies regionally and depends critically on the zonal versus meridional orientation of the AR moisture transport. Thus, extreme ARs with larger IVT do not always lead to strong winds or wind speeds at the surface, although they may cause extreme precipitation^8^.

AR-induced extreme temperature patterns are sensitive to AR threshold choice, with extreme AR temperatures showing widespread warming trends across most European regions^57^. To examine the thermal impacts of landfalling ARs, daily temperature values exceeding the 10^th^ percentile at each AR landfalling location were analysed (Fig. 11). The 10^th^ percentile temperature threshold exhibits a pronounced south-north gradient, ranging from 7 $^{\circ }$C in southern Europe to $-20$ $^{\circ }$C in northern (subpolar) Scandinavia (Fig. 11a). Mean temperatures above this threshold are consistently warmer, peaking at 10 $^{\circ }$C in the south and remaining below 0 $^{\circ }$C over Scandinavia (Fig. 11b). ARP levels significantly influence temperature anomalies, with the magnitude and spatial pattern of temperature responses varying systematically with detection threshold. Both “low” and “high” confidence AR footprints produce similar spatial patterns but with distinctly different intensities: substantial warming of 3–4 $^{\circ }$C occurs in northern regions. In contrast, 1–2 $^{\circ }$C cooling affects southern areas, both relative to the 10^th^ percentile baseline (Fig. 11c,d). Critically, “extreme” ARs consistently produce temperature anomalies approximately 1 $^{\circ }$C more intense (for both cooling and warming) compared to ARs with higher mapping uncertainty, demonstrating the threshold-dependent nature of AR thermal impacts. This pattern reveals that “extreme” ARs generate more pronounced cooling over Europe and Scandinavia than ARs with higher uncertainty in mapping, with the cooling effect being most evident in the higher-confidence detection category/regions.

The relationship between AR detection parameters and temperature remains generally weak but regionally variable (Fig. 11e,f). Negative correlations predominate in Eastern Europe and the Mediterranean, while positive correlations are primarily found in narrow coastal regions of Europe and the UK. These cooler temperatures can systematically lower the ARP, creating a feedback mechanism between thermal conditions and AR identification. In contrast, increased IVTs consistently drive warming over landfalling locations across the UK, Europe, and Scandinavia (Fig. 11g,h). This warming response proves more pronounced for larger ARs with higher uncertainty compared to narrow, “high” confidence ARs, and the effect intensifies northward, being most significant in Scandinavia relative to the UK and central Europe. Extreme AR temperatures exhibit clear warming trends across most regions, with the magnitude and spatial extent varying depending on the AR detection criteria. Larger ARs (with greater mapping uncertainty) show annual temperature increases of 0.01–0.02 $^{\circ }$C y^-1^, with statistically significant warming concentrated in Iberia, coastal Europe, Eastern Europe, Sweden, and the UK (Fig. 11i).

The spatial pattern of warming shifts dramatically when using “high” confidence (ARBT) criteria. While the overall warming signal persists, these temperature increases become more localised along the western European coast, the Iberian Peninsula’s Mediterranean coast, the Alps, and northern Scandinavia, where annual warming can exceed 0.04 $^{\circ }$C y^-1^ (Supplementary Figure S4). This demonstrates that the choice of AR threshold fundamentally alters both the magnitude and geographic distribution of observed temperature trends. These temperature results strongly corroborate the earlier findings for precipitation and wind patterns (Figs. 7, 8, and 10), highlighting the robust coupling between surface temperature and wind speeds during AR landfall events. The systematic relationship between AR detection confidence, thermal anomalies, and associated meteorological impacts emphasises that AR-induced surface temperatures are indeed increasing across most regions. Still, the specific patterns and magnitudes depend critically on the AR threshold choice employed in the analysis. The key finding is that AR-induced temperature impacts are threshold-sensitive, with temperatures exhibiting consistent warming trends across Europe; however, the spatial patterns and magnitudes vary significantly depending on the AR detection criteria applied.

## Discussions

Orography and regional climate effects play a crucial role in determining how ARs influence weather extremes at a specific location. At the same time, it is essential to reduce uncertainty in detecting ARs to better understand their impact on local weather extremes. Reduced structural uncertainty in AR mapping can help narrow down the area most affected by the AR landfall by eliminating errors in mapping the dimensions and lateral boundaries of the AR. It is essential to note that the heuristic nature of ARBT can’t be ignored when qualitatively estimating the impacts of AR over land (Figs. 5 and 6). This is because the characteristics of ARBT mapped using the TECA-BARD model vary depending on the selected probability and may show differences within the study region^29^. Moreover, the IVTs, the area affected by the AR landfall and associated meteorological variables, show a large difference between ARP and ARBT (Figs. 1 and 2).

An increase in AR frequencies and intensities, leading to “high confidence ARs” embedded in winter storm paths, could be causing the observed spatial “dipole patterns” in AR mean and standard deviations during half-years over the Euro-Atlantic region (Fig. 3). A predominant effect that could lead to seasonal differences is the positive phase of ENSO, which is associated with more ARs globally and near the equator^58^. Guan and Waliser^30^ found a strong coherence between AR changes and ENSO in the northern hemisphere, particularly in winter. During the positive phase of ENSO, ARs tend to move closer to the equator, and more areas experience an increase in AR frequency during the winter season, thus causing these patterns. The changing regimes in ENSO, NAO, blocking, jet stream and Rossby wave undulations could further enhance these biases in different seasons and half-years^58^. Particularly, the ENSO and NAO effects are more pronounced and persistent during the autumn and winter months, lasting from September to February.

The differences in half-years are also attributed to the permissive and restrictive nature of tropical filters used by AR detectors. There is a connection between strong El Niño (ENSO+) events and positive IVT anomalies in both the tropics and mid-latitudes^59^. The impact of these anomalies on the detection of ARs depends on the values of the tropical filter in the AR detectors. Among the 1024 AR detectors in the TECA-BARD model, detectors with high tropical filter (permissive) values would aggressively filter out positive IVT anomalies in the tropics, resulting in only the higher-than-average IVT in the mid-latitudes, which would affect AR detections. This would lead to an increased number of ARs during El Niño (ENSO+) events. AR detectors with low tropical filter (restrictive) values would not eliminate the positive anomaly in the tropics. As postulated by O’Brien et al.^29^, this increases the chance of the high IVT zones in the mid-latitudes being connected to the high IVT zones in the tropics, resulting in fewer but larger-than-average ARs during El Niño events. The AR detections over the high IVT zone of the eastern U.S. and along the Gulf Stream could be affected by the restrictive and permissive nature of detector samples, which tend to show differences in ARPs during the half-years.

Similarly, moderate and low “confidence” ARs occur frequently in inland Europe along the great European plains. The larger difference in the frequency of ARs over a region, for a given number of AR detections, arises from their area/footprint and the width of their lateral boundaries. The ARBT with lower structural uncertainty in mapping has narrow boundaries (lower area footprint), whereas the ARP with larger uncertainty has a broader reach. Another reason for frequency differences (Fig. 4) could be AR’s paths when considering ARP as a whole and only “high” confidence ARs. High “confidence” ARs embedded in the winter storm tracks move east and northeastward along coastal Europe and maintain high IVT contributed by both zonal and meridional flow due to the continuous supply of moisture from the adjacent ocean^60–62^. ARs moving more zonally towards inland Europe may lose their IVT (tends to lower ARP) after landfall due to moisture cut off from the ocean. Thus, the lower IVTs can lead to low “confidence” in TECA-BARD detection. However, with favourable conditions and less orographic attenuation, weaker and moderate ARs (with lower ARP) driven by more zonal flow could move zonally towards the northeast to reach more inland and eastern European areas. This persistence in inland moving ARs causes ARP to have a larger frequency over these areas than ARBT. More specific reasons for these differences need to be explored further.

ARs often bring increased cloud cover, which can trap outgoing longwave radiation from the Earth’s surface through water vapour feedback, cloud-radiation processes and could lead to warmer winters^57^. This would result in less energy loss from the surface, mainly due to increased downward longwave radiation and reduced upward sensible heat, leading to a warming effect, particularly during nighttime^63,64^. Strong winds can mix air masses vertically and horizontally, potentially bringing advected warmer air to the surface from aloft or redistributing air masses in a way that leads to surface warming (Fig. 7b). Additionally, systematic studies highlight the significant role of ARs in the meridional transport of atmospheric latent heat, affecting climatic parameters in subpolar regions, with a notable impact in the northern hemisphere due to the absence of constant blocking effects^65,66^. The decrease in temperature over the Iberian Peninsula could be attributed to the dominant effect of reduced direct irradiance due to increased cloud cover, rather than anomalous downwelling longwave radiation. Enhanced precipitation from ARs brings substantial cloud cover and reduced solar radiation, leading to cooler daytime temperatures over land. Intense AR precipitation can increase soil moisture, contributing to evaporative cooling and lowering surface temperatures. “High” confidence ARs in the winter are more intense (and frequent) and bring precipitation in the form of snowfall, which can reduce the surface temperatures. Moreover, the fierce ARs developed from the strong geostrophic flow and thermal wind are followed mainly by the cold front, whose cold air can lower the temperature over AR landfall in the mid-latitudes (Figure 7e). The less persistent of intense ARs (ARBT) over the region due to their tendency to higher precipitation may also impact their relationship with temperatures.

These relationships among AR-induced meteorological variables can be further explained by the seasonal AR flavours (families) and orientation^50,52^, which can advect cooler air from the north when dominated by the meridional IVT component. In some cases, strong winds may follow the passage of a cold front, clearing the sky. Clear skies at night allow for more effective radiative cooling of the Earth’s surface, resulting in lower temperatures, especially during northerly winds. Strong vertical stability can be established when surface temperatures are colder than the surrounding atmosphere, due to the warmer atmosphere over a colder surface. This creates more stable conditions that are opaque to momentum transfer from the AR jet to the surface. On the other hand, when the atmosphere is colder than a warmer skin surface temperature, it creates the opposite effect and is conducive to strong surface winds^47^. This could further lead to reduced radiative cooling from the surface (Fig. 7c,f).

Though ARBT ($\ge$0.67) can represent the areas with larger IVTs over Europe and the UK, ARs with selected thresholds show moderate correlations with precipitation over the UK and coastal Europe. Additionally, it may not be sufficient to consider extreme IVTs as the sole explanation for precipitation peaks in the region. It is also essential to assess local conditions, seasonal AR flavours, topography, and the impact of other meteorological variables during extreme AR landfall. The higher frequency of strong ARs during winter over the region can further enhance these effects, leading to uncertainty in gauging AR-induced river discharge and flooding. While the frequent occurrence of ARs in a winter season can also shorten the length of the season due to enhanced snow loss and temperature rise (Supplementary Figure S4)^67,68^; persisting uncertainty in AR mapping could enhance these effects.

Unstable atmospheric conditions contribute to the lower correlations between extreme wind speeds and extreme precipitation during the landfall of intense ARs (ARBT). Turbulence can enhance surface winds independently of precipitation patterns, leading to stronger surface winds associated with ARs under weaker vertical stability conditions in the atmospheric layer. The temperature difference between the surface and the low-level jet core increases under such weaker conditions, resulting in turbulence and mixing, which can increase surface winds without necessarily correlating with the intensity of precipitation^47^. The ARs over the Euro-Atlantic region seem to be flavoured towards wet or windy and warm (Fig. 7 and Supplementary Figure S1). The study by Pagano^47^ found that the AR cases within the top and bottom 25% of near-surface stability show that extreme surface winds (gale or higher) are more likely to occur in unstable conditions than in stable conditions. Specifically, during weak and strong IVT conditions, extreme surface winds are more likely in unstable conditions (5.3% and 14.7%, respectively) than in stable conditions (0.58% and 6.15%). This suggests that the atmospheric stability near the surface plays a significant role in extreme wind speeds during AR landfall, which can lead to a mismatch between the intensity of wind speeds and precipitation^8,47^.

AR frequency, intensities, paths, orientation, orography, and gradual loss of energy by ARs before reaching deep inland (causing negative annual trends) are some of the factors that contribute to annual changes in wind speeds. Altering lower atmosphere vertical stability driven by the influence of local climate patterns also plays a key role in these annual trends. Many of the above aspects require a thorough investigation using case studies and modelling, supported by observations from various locations, which are beyond the scope of the current work.

## Summary and conclusions

Numerous studies have examined uncertainty in AR studies, including uncertainty linked with reanalysis and climate models and uncertainty in AR detectors^11,28,30,35,69^. Studies based on ARTMIP have found that AR detection uncertainty is more extensive than anticipated, comparable to model uncertainty in future climate simulations^7,11,27–29^. TECA-BARD is one of the methods used to map uncertainty in AR detection using multiple priors (1024 detectors) designed through the Bayesian model applied to the global AR detections from 8 atmospheric scientists. While the method performed well globally across different regions, its accuracy decreased in the Euro-Atlantic region, with a value of 0.6, which challenged the method and highlighted the complexity of the AR study in the region. A narrow ocean area of the northern Atlantic with high IVTs makes studying ARs difficult, despite various climate modes driving multiple dynamic and thermodynamic phenomena in the region.

While the developers tested TECA-BARD using the number of AR detections over different regions globally, there are numerous aspects of AR-related research for which TECA-BARD could be useful. First, we implemented the algorithm on a regional scale to research AR variability, predictability, and impacts on meteorological extremes in the observational record, using high-resolution, long-term (1940–2022) and significantly improved ERA5 data. Although the heuristics are not entirely removed in this approach, the parametric dependencies on IVT, length-to-width ratio, tropical filter, minimum area of ARs, etc., can be reduced using the model. Despite providing uncertainty in the form of AR probabilities, the model allows users to choose a probability of their choice to study ARs concerning the study area. The developers used a default choice of 0.67 probability (ARBT) to map AR areas globally, and they suggest that this would reduce the uncertainty in AR mapping, which is analogous to setting a threshold for IVT^30^.

In addition to ARBT, we use the entire probability (ARP) and different deciles (at 10% ARP increments) to investigate the AR structural uncertainty on meteorological variables over the Euro-Atlantic region. The following are some key findings from the study.

- AR intensity estimation, area of impact, and precipitation magnitudes are affected by uncertainty in AR structure (Figs. 1 and 2). This uncertainty also affects the spatial (mean paths and frequencies over land) and temporal (half-year and annual) characteristics of ARs, IVT, and its components (Figs. 3 and 4).
- The selected probability is critical in quantifying an AR’s impact over the landfalling area in Europe. The selected ARP decile strongly guides the magnitude of precipitation, wind speeds, daily temperature and annual trends/changes during AR landfall (Figs. 5 and 6).
- ARP magnitude is strongly coupled with IVT magnitude and its components. Thus, there are large differences between ARPs over land (lower IVTs) and the ocean (higher IVTs), increasing the uncertainty over land.
- While ARP can explain a significant portion of precipitation and wind ($\ge$ 90%) and temperature extremes ($\ge$ 10%) with minimal bias, ARBT and the associated IVT also play a crucial role in these extremes. However, it is uncertain whether higher IVTs consistently lead to AR-induced extremes over land. In favourable atmospheric conditions, even lower IVTs (resulting in less ARP) may trigger compound extremes on land.
- Structural uncertainty in AR mapping causes significant differences in AR frequency across the UK, coastal and North Eastern Europe, and southern Sweden.
- Annual changes in precipitation and wind speeds from ARs are more affected by AR structural uncertainties than temperature. These detection uncertainties impact inland Europe and Scandinavia more than coastal Europe and the UK (Figs. 9i, 10i, 11i; Supplementary Figures S2, S3c and S4).
- “Wet” ARs cause wind anomalies, and “windy” ARs lead to warmer temperature anomalies (Fig. 7). Temperature anomalies during the “wet” ARs are more sensitive to the AR structural uncertainty. While AR-induced concurrent extremes over the region are either “wet” or “windy and cold”, not every extreme AR can lead to compound extremes (Supplementary Figure S1).
- A marked gradient in AR-induced extremes was observed, with correlations for precipitation ranging from 0.2 in the north and east to 0.4 in the south and west of Europe. For wind extremes, correlations vary from 0.35 in the west to 0.10 in the south and east.
- The temperature gradient is stronger north–south than west–east, with the highest correlation of 0.55 in the north and the lowest at 0.25 in southern Europe. This pattern is consistent in Scandinavia, regardless of AR mapping uncertainty, but with landfalling “high” confidence ARs, the correlation magnitudes are halved.
- Explosive storms moving east or northeastward may lead to high confidence ARs along the UK and Europe due to persistent moisture availability. Eastward-moving storms may weaken after landfall but could still produce weaker/moderate ARs over Europe.
- Warm, moist air from ARs may raise temperatures in the UK, eastern Europe, Iberia, and southern Sweden, contributing to annual temperature increases. Additionally, ARs have also led to greater annual precipitation in the UK, France, the Mediterranean coast, and Northern Sweden.
- Eastward-moving ARs may lead to extreme wind speeds across the UK and Europe. However, extreme wind speeds are declining in most parts of the study region (Fig. 10 and Supplementary Figure S3).

ARP landfalling area is affected by a selected ARP, and the intensity of IVTs causes spatial and temporal patterns in AR characteristics. An equator-ward shift of ARs due to strong climate modes (ENSO+) and changes in their paths during winter may lead to these seasonal differences in AR patterns forming a “dipole” over the Atlantic. The positive correlation between wind and precipitation, as well as temperature anomalies, suggests that stronger winds accompanying ARs may enhance moisture transport, leading to warmer temperatures and increased precipitation in the region. The negative correlation between temperature and precipitation anomalies suggests that temperatures tend to be relatively lower in cases where precipitation is particularly intense, potentially due to processes such as evaporative cooling or cloud cover limiting daytime warming.

Using a 60% threshold (D60) from TECA-BARD is the minimum needed to capture AR impacts over the Euro-Atlantic region. Different ARP and ARBT thresholds show large variations in correlations between precipitation, wind speed, and temperature anomalies (Fig. 7). Higher-confidence ARs exhibit stronger correlations across larger areas, with AR-induced precipitation and temperature anomalies showing a strong correlation with wind speed anomalies in the UK and Western Europe. During high-confidence AR landfalls, intense precipitation causes temperature drops except over Iberia and parts of the UK, indicating that AR mapping uncertainty affects the location and magnitude of extreme weather events. While annual AR area and IVT trends show minor changes beyond D10 (Fig. 5a,b), ARP decile selection significantly impacts annual trends in AR-induced meteorological variables (Fig. 5c–e). AR-induced precipitation and wind speed trends are more sensitive to AR dimension uncertainty than surface temperatures. The causes of these large changes in meteorological parameter trends, despite small IVT and dimensional changes, remain unclear and require further investigation into climatic conditions, dynamics, and the influences of climate modes on regional AR circulation patterns.

### Limitations of the present study

Though using TECA-BARD reduces the underlying uncertainty in mapping ARs and studying their impacts over land, there are certain limitations in designing the model as a whole and implementing it in the present study. We were not conscious of the biases/production errors in the input data used from ERA5 and E-OBS. No special emphasis is given to the seasonal and semi-annual analysis in the current study. A seasonal study (including winter and summer half-years) might produce different results. TECA-BARD is based on input from 8 experts drawn from global MERRA-2 3-hourly data of only 2 years, without special emphasis on seasonal and regional outlook. This may limit the range of uncertainty that TECA-BARD can explore in studying Euro-Atlantic (regional) ARs due to the sampling bias and limited sample size, which could potentially bias the detector toward a particular definition of AR. Thus, the results are valid on the assumption that there will be no strong bias in the regional implementation of TECA-BARD compared to its global usage.

However, the region analysed in this study (0–80$^{\circ }$ N, 100$^{\circ }$ W–25$^{\circ }$ E) is large enough to permit variation in the range of IVTs, making them comparable to global IVT magnitudes. On the other hand, we implemented the model on higher spatial (ERA5) data and lower temporal (6-hourly) resolutions in the current study. No account of climate modes, such as ENSO+, is considered in the development of TECA-BARD. Thus, we limit the study only to the spatial variability of meteorological extremes in relation to the detection uncertainty in the study period; the model’s performance during strong climate mode scenarios will be explored further. No special attention is given to seasonal biases in the development of TECA-BARD. As AR characteristics vary between summer and winter across regions like the Euro-Atlantic area (Fig. 3), tailored solutions are required to counteract the bias.

Future work includes an independent study designed to test the TECA-BARD’s sensitivity at the regional level and on different temporal scales, compared to its global application. The same can be extended to study changes in AR dynamics and impacts on past and future climates. A similar approach can be used to study the impact of different climate modes, changing atmospheric dynamics and thermodynamics on Euro-Atlantic ARs, where parametric uncertainty can have a large effect on data analyses. A study is designed to develop a machine-learning model to investigate AR-induced hydro-meteorological extremes and their connections, and to understand the physical factors (parameterisation) impacting them.

## Supplementary Information

Supplementary Information.

## Data Availability

All the data used in the study are freely available online from corresponding data sources cited in the article https://cds.climate.copernicus.eu/. The data supporting this study’s findings are available on request from the corresponding author.

## Code availability

All codes used in this study are available on request from the corresponding author. TECA package, installation and usage instructions are available freely at https://teca.readthedocs.io/ and https://github.com/LBL-EESA/TECA.
