# Supplementary material for: Structural uncertainty in mapping Euro-Atlantic atmospheric rivers obscures understanding of associated meteorological extremes
Source: Sci Rep. 2025 Sep 29;15:33325. doi: 10.1038/s41598-025-19685-1 (PMC12479941; doi:10.1038/s41598-025-19685-1)
Supplement: Supplementary file 1 — Supplementary Information. [file 41598_2025_19685_MOESM1_ESM.pdf]

Structural Uncertainty in Mapping Euro-Atlantic Atmospheric Rivers Obscures Understanding of Associated Meteorological Extremes

Venugopal Thandlam[1,2,3], Anna Rutgersson[1,2], and Erik Sahlee[1]

1. Air, Water and Landscape Science (LUVAL), Department of Earth Sciences, Uppsala University, Uppsala, Sweden
2. Centre of Natural Hazards and Disaster Science, Uppsala University, Uppsala, Sweden
3. The Center for Environment and Development Studies Research Forum, Uppsala University, Uppsala, Sweden

Supplementary material:

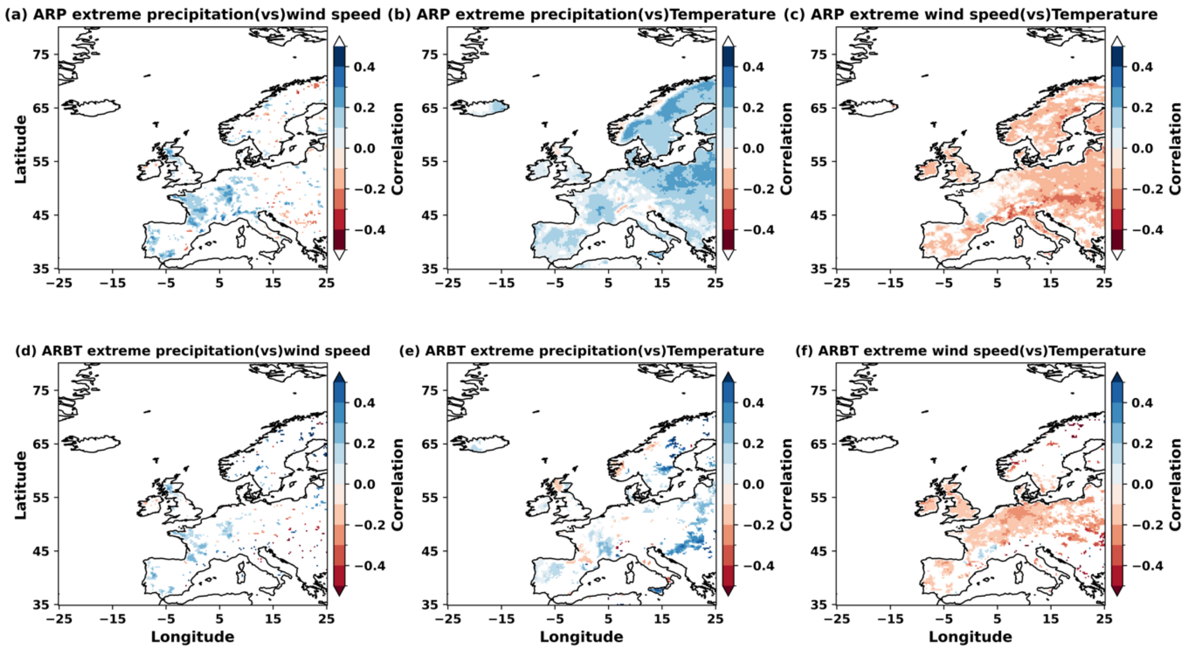

Figure S1: The spatial variability of PCCs of meteorological extremes for ARP (row 1: a, b, and c) and ARBT (row 2: c, d, and e). PCCs are computed among the daily precipitation, wind speeds (>90th%) and TN (>10th%). All PCCs shown are significant at 95% and above. The figure is generated using Python v3.12.4.

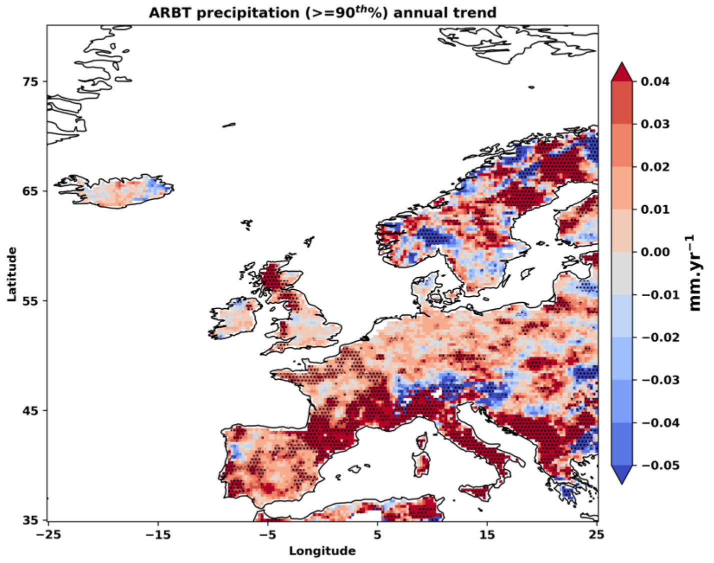

Figure S2: Annual precipitation trend (mm/yr) associated with ARBT. Overlaid dots show the area with significant trend (95% and above) values. The figure is generated using Python v3.12.4.

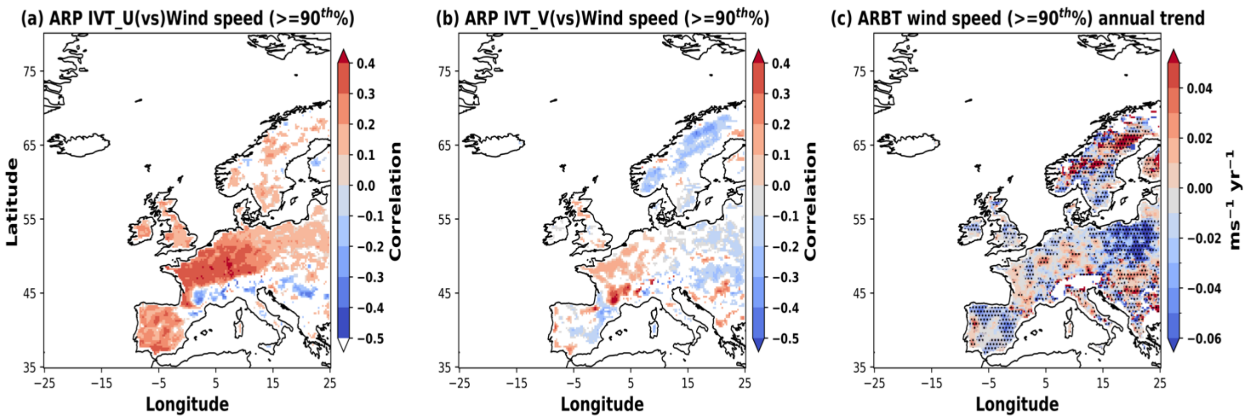

Figure S3: ARP PCCs between daily wind speed and (a) daily IVT_U and (b) IVT_V. Only significant (at 95% and above) PCC values are shown. The figure is generated using Python v3.12.4.

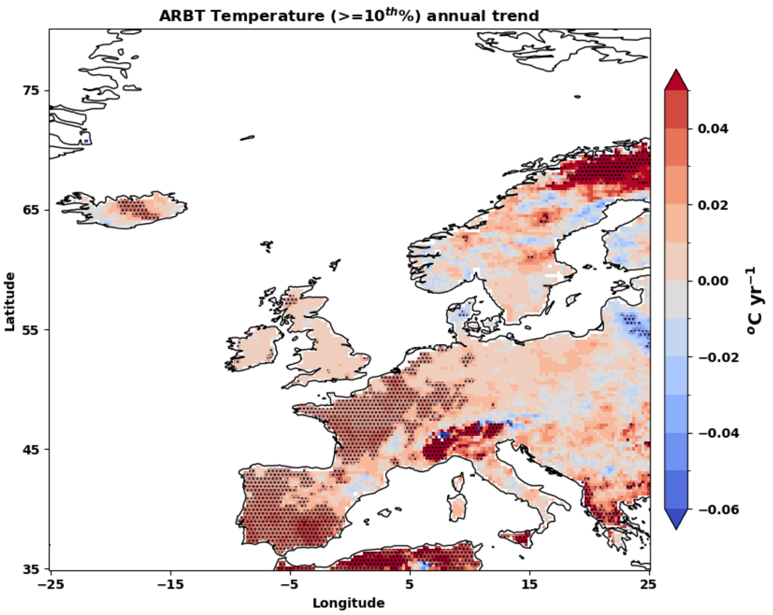

Figure S4: Annual trend of ARBT TN (>=10th%). Areas with a significant trend (95% and above) are marked with dots. The figure is generated using Python v3.12.4.
